# Therapeutic effect of *Lepidium peruvianum Chacon* mediated silver nanoparticles on fertility in adult male Sprague-Dawley rats

**DOI:** 10.1039/d5ra05660h

**Published:** 2025-09-17

**Authors:** Ameer Hamza, Muhammad Babar Taj, Merfat M. Alsabban, Amal Marzoqi Altowerqi, Sarwat Jahan, Aysha Afzal, Saad Alghamdi

**Affiliations:** a Department of Zoology, Quaid-i-Azam University Islamabad 45320 Pakistan; b Institute of Chemistry, The Islamia University Bahawalpur 63100 Pakistan dr.taj@iub.edu.pk; c Department of Chemistry, College of Science, University of Jeddah Jeddah 21959 Saudi Arabia; d Department of Physics, University of Taibah 42353 Saudi Arabia; e Department of Clinical Laboratory Sciences, Faculty of Applied Medical Sciences, Umm Al-Qura University Makkah Saudi Arabia

## Abstract

Male infertility is a growing global health issue, accounting for 50% of cases, and highlighting the need for innovative treatment solutions. This study presents a phyto-mechanochemical approach for synthesizing silver nanoparticles (LP@AgNPs) using *Lepidium peruvianum Chacon* (LP). The LP@AgNPs were characterized using physiochemical techniques. Adult male Sprague Dawley rats (*n* = 15) were divided into three groups and treated orally for 28 days with distilled water, LP powder, or LP@AgNPs. The UV-visible spectrum exhibited a pronounced surface plasmon resonance peak at 415 nm, and morphological analysis indicated a crystalline structure characteristic of a typical face-centered cubic (FCC) lattice with an average crystallite dimension of 11.388 nm, as confirmed by powder X-ray diffraction (PXRD). The LP@AgNPs displayed a zeta potential of −41.18 mV, indicating high stability and low aggregation potential. The administration of LP@AgNPs induced significant histomorphological and morphometric changes in reproductive organs supporting enhanced spermatogenesis, spermatozoa maturation, and semen quality, thereby improving the fertility potential of male rats. The biochemical profile exhibited a non-significant change in follicle-stimulating hormone (*P* = 0.195), a significant reduction in luteinizing hormone (*P* = 0.001), and a marked increase in testosterone (*P* = 0.0001). However, oxidative stress biomarkers and antioxidant enzyme concentrations remained unchanged following LP and LP@AgNPs administration. Overall, these findings suggest that LP@AgNPs improve fertility and have the potential to act as a promising therapeutic option for male reproductive health.

## Abstract 2

The study investigates male infertility by synthesizing silver nanoparticles (LP@AgNPs) using *Lepidium peruvianum* Chacon. LP@AgNPs showed significant improvements in reproductive tissues, spermatogenesis, and semen quality in Sprague Dawley rats.

## Graphical abstract

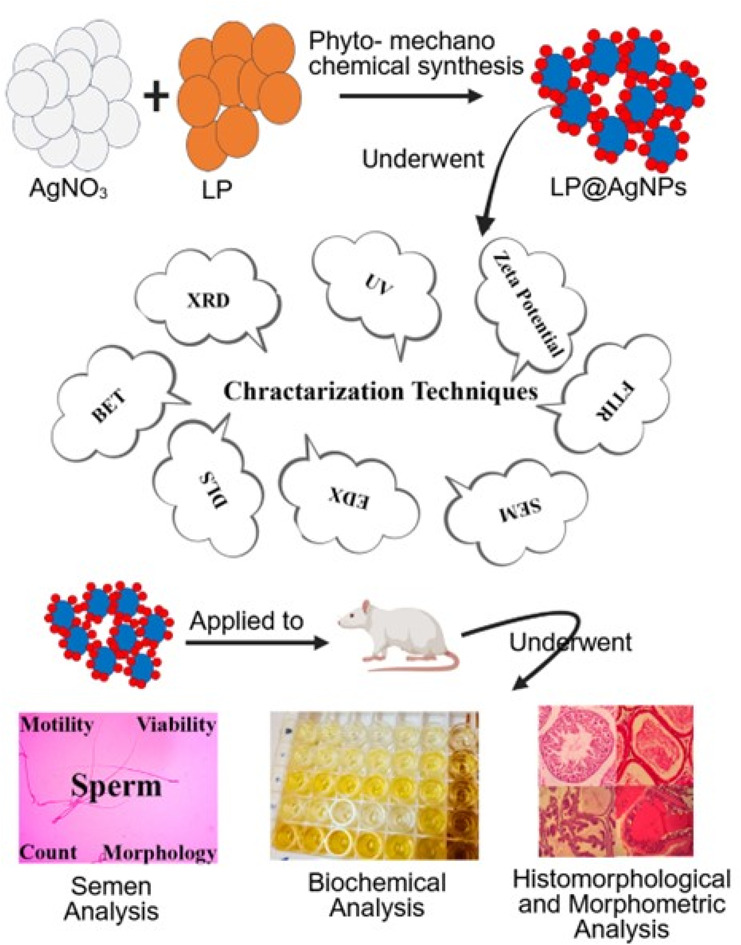

## Introduction

Male infertility is a significant global health issue, affecting approximately 8–12% of couples worldwide, with male factors contributing to 50% of cases.^1^ A man is considered infertile if pregnancy does not occur after one year of unprotected intercourse. While 60% of couples conceive within six months, 90% do so within a year.^2–4^ In the U.S., 15% of couples face infertility,^5^ and in Pakistan, male-related infertility accounts for up to 21%.^6^ Over the last four decades, sperm quality and count have declined, leading to one in twenty men experiencing infertility.^7,8^ Poor semen quality, including issues like azoospermia, oligospermia, limited motility, and abnormal morphology,^9^ is a key indicator of male infertility.

Despite advances in understanding the cause of male infertility, conventional treatments address only a limited proportion of cases.^10^ In contrast, assisted reproductive technologies (ARTs), including *in vitro* fertilization (IVF),^11,12^ intracytoplasmic sperm transfer (ICSI)^13^ artificial insemination (AI),^14,15^ intrauterine insemination (IUI)^16,17^ and foster adoption^18,19^ are often considered as alternatives. However, these methods are associated with limited success rates accompanied by complications and high costs, highlighting the need for more effective and accessible treatments for male infertility.^20^

Nanotechnology is significantly impacting reproductive physiology, particularly in infertility research.^21–23^ The synthesis of nanoparticles (NPs), especially silver nanoparticles (AgNPs), from biological materials is a key area of focus. AgNPs were used in this study due to their well-documented antibacterial, antioxidant, anti-inflammatory, and unique physiochemical properties.^24^ Previous studies have demonstrated that they have the potential to improve reproductive health in both males and females. *C. zeylanicum-*derived AgNPs regulated sex hormones in females suffering from polycystic ovarian syndrome, garlic-derived AgNPs improved semen quality, *P. fulva-*based AgNPs preserved uterine histology and modulated estrogen and progesterone levels, and *N. sativa*-derived AgNPs alleviated chromium-induced reproductive toxicity in male rats. The plant-based synthesis of AgNPs offers several advantages, including cost-effectiveness, biocompatibility, scalability, low toxicity, and environmental friendliness.^25–29^ Additionally, the high surface area-to-volume ratio of AgNPs allows efficient interaction with biological molecules, enhancing their drug delivery capacity and therapeutic efficacy.^30^ Phyto-mechanochemical synthesis is noted for its scalability, safety, and cost-effectiveness.^31,32^ Plant-derived molecules, such as alkaloids, sugars, and polyphenols, play a crucial role in the bio-reduction of silver ions, influencing the formation and properties of NPs.^33^ Additionally, the use of different bio extracts allows for precise control over the morphology and size of NPs, thereby enhancing their versatility and applications.^34^

Among natural resources, *Lepidium peruvianum Chacon* (LP) is a cultivated plant species in the Andean region. It was originally deposited in the Herbarium of San Marcos University in Lima, Peru, with records dating back to 1843 and 1990.^35^ Recent research has shown distinct differences in taxonomy, visual appearance, phytochemical profiles, and DNA sequences between LP and *L. meyenii*.^35^ The research indicates that LP possesses all the characteristics unique to this historically documented herb, which is grown in the Andean highlands. This may be linked to phytochemicals (chemical composition of macamides, polyphenols, glucosinolates, and alkaloids), which contribute to color-specific physiological effects, including the modulation of sex hormones, sperm quality, and sexual functions.^36,37^ These aspects were confirmed in recent clinical studies, recognizing their relevance for contemporary dietary, therapeutic, and health benefits.

Recent investigations into the ameliorative effects of AgNPs have primarily focused on their impact on individual organs, typically within a limited range of parameters (62–65). Although these studies yield valuable insight, they often fail to comprehensively assess reproductive system dynamics in response to drug administration, particularly the morphological and functional interrelationships between the primary and accessory sex glands. In contrast, our study employs a comprehensive approach by synthesizing novel LP@AgNPs through a phyto-mechanochemical method and systematically investigating their beneficial effects on the reproductive system of adult male Sprague-Dawley rats.

To the best of our knowledge, there is currently no published research on the LP-mediated phyto-mechanochemical synthesis of AgNPs. This study aims to synthesize and evaluate the therapeutic potential of LP@AgNPs on male reproductive functions, focusing on structural, biochemical, and semen quality parameters in adult male Sprague Dawley rats. The main objectives of this study are to: characterize the LP@AgNPs; evaluate their impact on male reproductive health by observing histopathological changes in reproductive and vital organs (testes, epididymis, prostate, seminal vesicles, liver, kidney, and heart); quantify changes in reproductive hormones (follicle stimulating hormone (FSH), luteinizing hormone (LH), and testosterone (T)); analyze antioxidant enzymes and oxidative stress biomarkers; and determine their effects on semen quality, including sperm count, viability, motility, and morphology.

## Methodology

### Plant collection

The LP was collected from a local pinsar store and mechanically ground into fine powder. An aqueous extract was synthesised by adding 10 g of the LP powder to 100 mL of distilled water (dH_2_O).

### Chemicals

Iron(iii) chloride (Sigma-Aldrich; CAS number:7705-08-0), ammonium hydroxide (Sigma-Aldrich; CAS number:1336-21-6), chloroform (Sigma-Aldrich; CAS number: 67-66-3), sulfuric acid (Sigma-Aldrich; CAS number:7664-93-9), ethanol (Sigma-Aldrich; CAS number:64-17-5), potassium ferricyanide (Sigma-Aldrich; CAS number:13746-66-2), sodium hydroxide (Sigma-Aldrich; CAS number:1310-73-2), glacial acetic acid (Sigma-Aldrich; CAS number:64-19-7), silver nitrate (Sigma-Aldrich; CAS number:7761-88-8), hydrogen peroxide (Sigma-Aldrich; CAS number:7722-84-1), NADPH (Sigma-Aldrich; CAS number:24292-60-2), reduced glutathione (Sigma-Aldrich; CAS number:70-18-8), glutathione disulfide reductase (Sigma-Aldrich; CAS number:9001-48-3), 1-chloro-2,4-dinitrobenzene (CDNB) (Sigma-Aldrich; CAS number:97-00-7), guaiacol (Sigma-Aldrich; CAS number:90-05-1), trichloroacetic acid (Sigma-Aldrich; CAS number:76-03-9), thiobarbituric acid (Sigma-Aldrich; CAS number:504-17-6), ascorbic acid (Sigma-Aldrich; CAS number:50-81-7), EDTA (Sigma-Aldrich; CAS number:60-00-4), eosin (Sigma-Aldrich; CAS number:17372-87-1), nigrosin (Sigma-Aldrich; CAS number: 8005-03-6), hematoxylin ((Sigma-Aldrich; CAS number:517-28-2). The chemicals used in this experiment were 99% pure (analytical grade).

### Phytochemical analysis

The phytochemical analysis of LP was performed to identify the bioactive constituents involved in green synthesis of AgNPs and assess their potential therapeutic and toxicological contributions.

### Test for saponins

4 mL of the LP extract was dissolved in 40 mL of dH_2_O and boiled. The mixture was stirred vigorously for 20 min. Two drops of olive oil were added, and the formation of white precipitates (PPTs) indicated the presence of saponins in the extract.^38^

### Test for tannins

The LP extract (1 mL) was diluted with 20 mL of dH_2_O, boiled, filtered, and treated with 6 mM FeCl_3_ solution. The brownish–green colour highlighted the presence of tannins in the LP composition.^38^

### Test for flavonoids

Two drops of NH_4_OH were introduced to 2 mL of the LP extract. 5 mL concentrated H_2_SO_4_ was added to the reaction mixture and mixed well. The yellow–brown PPTs indicated flavonoids in the extract.^38^

### Test for steroids

Salkowski's reaction confirmed the presence of steroids in the LP extract. 2 mg of dried powder was taken and shaken well with chloroform. H_2_SO_4_ was poured slowly along the walls of the test tube. The appearance of the red colour indicated the presence of steroids.^39^

### Test for alkaloids

A single drop of Mayer's reagent was added to 1 mL of the LP extract. White PPTs that emerged in the mixture confirmed the presence of alkaloids.^38^

### Test for anthraquinones

5 mL of the LP extract was treated with 1 mL of H_2_SO_4_ and 1 mL of NH_4_OH. The pink colour indicated the presence of anthraquinones.^38^

### Test for polyphenols

4 mL of C_2_H_5_OH was added to 1 mL of the LP extract. The mixture was heated for 3 min, and three drops of ferric cyanide were added. The colour of the mixture was altered to blue-green, which indicated the presence of polyphenols.^38^

### Test for coumarins

3 mL of 2.5 M NaOH was added to 2 mL of the LP extract. The appearance of yellow colour served as an indication of the presence of coumarins.^38^

### Test for cardiac glycosides

2 mL of glacial acetic acid (CH_3_COOH) was added to 5 mL of the LP extract, followed by the addition of 100 μL FeCl_3_ and 100 μL of concentrated H_2_SO_4_. In the middle of the solution, the presence of a brown ring confirmed the presence of cardenolides.^38^

### Synthesis of LP@AgNPs *via* phyto-mechanochemical milling

**Fig. 1:**
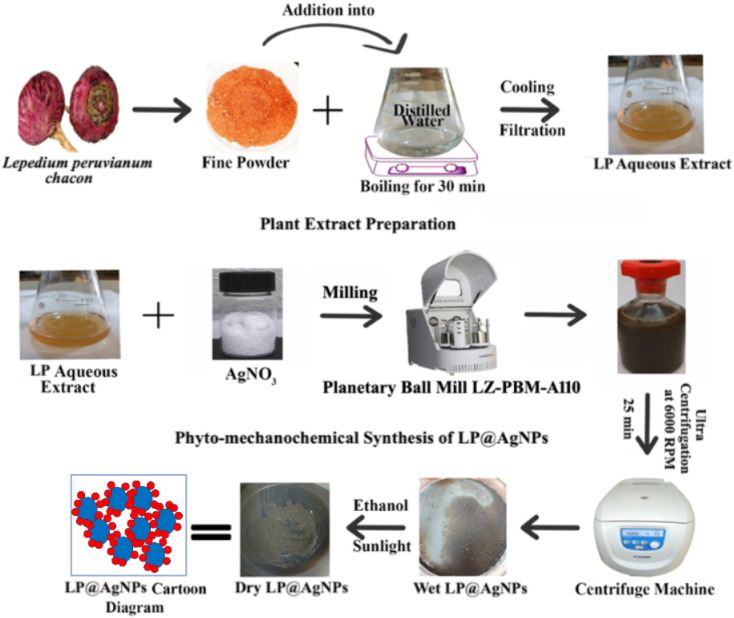
Phyto-mechanochemical synthesis of AgNPs: step-by-step LP extract preparation and NPs formation.

In a typical experiment, 2.00 g of AgNO_3_ was intimately mixed with 4.0 mL of concentrated LP extract and subjected to mechanochemical activation in a planetary ball mill (Model LZ-PBM-A110, Labozon, Canada). Milling was performed using tungsten carbide balls (Ø 10 mm) with a ball-to-powder mass ratio of 10 : 1, operated at a rotation speed of 500 rpm under an ambient air atmosphere. The grinding protocol consisted of a net milling time of 40 min, applied in a 5 min on/2 min off duty cycle (total elapsed time ≈ 63 min) to prevent thermal accumulation and minimize particle agglomeration. All experiments were conducted in a climate-controlled environment (24 ± 2 °C; 45 ± 5% RH) to ensure reproducibility. The resulting dark brown powder, designated LP@AgNPs, was recovered and subjected to centrifugation (10 000 rpm, 10 min), followed by successive rinsing with deionized water (dH_2_O) until no residual AgNO_3_ was detected (confirmed by the absence of nitrate peaks in UV-Vis spectra). The purified AgNPs were dried at 40 °C under vacuum and stored in amber vials until further characterization (Fig. 1).

### Characterization of LP@AgNPs

#### UV-visible spectroscopy

The formation of the LP@AgNPs was confirmed *via* UV-visible spectroscopy (Thermoscientific spectrophotometer UV-3100PC), indicating that AgNO_3_ was reduced to Ag. The data was collected in wavelengths ranging from 300 nm to 700 nm.

#### Fourier transform infrared spectroscopy

The reduction of AgNO_3_ into the LP@AgNPs by reducing agents found in the LP powder was determined using FTIR (Thermo Scientific USA FTIR, Model: Nicolet Summit Lite). The measurements were taken between 400 cm^−1^ and 4000 cm^−1^.

#### Powder X-ray diffraction

The crystallite size of the LP@AgNPs was analyzed using a Bruker D8 Advance PXRD system, equipped with a high-resolution Lynx Eye detector and a Cu radiation source.

#### Morphological studies

An SEM (Model: MIRA3 TESCAN) equipped with an EDX was employed to observe the surface morphology and elemental composition of the LP@AgNPs. The SEM images were captured at different magnifications.

#### Zeta potential (ZP) and dynamic light scattering (DLS)

The ZP was measured using a zetasizer (Litesizer DLS700). The same instrument was used to calculate the polydispersity index (PDI) and hydrodynamic size of the LP@AgNPs.

#### Brunauer–Emmett–Teller analysis (BET)

Nitrogen adsorption experiments were conducted at 196 °C using a Quantachrome NOVA 1200 (Odelzhausen, Germany). Before analysis, samples were preheated at 200 °C for 17 h. The specific surface area of the solid material was calculated using the BET method, and the Barrett–Joyner–Halenda (BJH) technique was employed to assess the porosity of the NPs.

#### Animals

Fifteen male Sprague-Dawley rats, randomly selected (75–95 days old), were obtained from the primate facility at the Department of Zoology, Quaid-i-Azam University, Islamabad. The animals were acclimatized in the animal house for seven days before the treatment. During the experimental period, they were housed individually in isolated stainless steel cages under controlled conditions (22 ± 2.0 °C, 50–60% relative humidity, and a 14 : 10 h light–dark cycle). Each animal received standard laboratory feed (20 g per animal per day, with unlimited access to water). All the animals received 1 mL dH_2_O through oral gavage during the acclimatization period. All treatments were administered orally at 10 am. daily to minimize circadian variations. The gavage was performed using a 6 mm diameter bird feeding tube, which was discarded after every use to prevent cross-contamination. The Bioethical Committee of Quaid-i-Azam University, Islamabad, approved all the studies (reference#BEC-FBS-QAU2024-625).

#### Acute toxicity evaluation

For the selection of the experimental dose for LP@AgNPs, an acute oral toxicity study was conducted. Four animals were each administered a single dose of LP@AgNPs at 250, 500, 750, and 1000 mg kg^−1^ body weight. The animals were continuously monitored for behavioral and physiological abnormalities throughout the study. On the 14th day, the dissection was performed to assess the health of the internal organs.

#### Experimental design

Animals (*n* = 15) were divided into three groups, and their body weights were monitored daily. The control group (*n* = 5) received 1 mL of dH_2_O per rat per day. The second and third treatment groups received the LP suspension and LP@AgNPs (200 mg kg^−1^ body weight per day) for 28 days. On the 29th day, dissections were performed to collect blood and organs for further experimentation. The weights of the reproductive organs were recorded for each animal. The schematic diagram of the experimental design and representative macroscopic images showing morphological changes in the animals are shown in Fig. S1 and S2. Reason for selection of dose (200 mg kg^−1^): Long-term oral exposure studies in adult rats have frequently included doses up to 200 mg per kg per day to evaluate AgNP toxicokinetics over extended periods. For instance, a 90-day sub-chronic study administering PVP-coated AgNPs at 50, 100, and 200 mg per kg per day measured silver accumulation, hematology, serum biochemistry, and tissue histopathology—demonstrating that this dosage is established in toxicity evaluation frameworks.^40^ In acute pharmacological investigations, plant-derived AgNPs administered at doses up to 2000 mg kg^−1^ did not result in mortality, underscoring a broad therapeutic window and justifying the use of 200 mg kg^−1^ as a moderate yet potent dose for functional assessments.^41^ By selecting 200 mg kg^−1^, we aimed to achieve a pharmacologically active dose that reflects both *in vivo* relevance and caution, lying within an extensively studied range (50–200 mg kg^−1^) while avoiding high-dose toxicity extremes (*i.e.*, >1000 mg kg^−1^).

#### Histology

The body organs were fixed in 10% formalin, dehydrated, paraffin-embedded, and sectioned using a microtome (Thermo Shandon Finess 325). The sections (5 μm thickness) were stained with hematoxylin and eosin (H&E). The slides were examined under a light microscope (Olympus CX41, attached to a Tucson USB 2.0 H series Camera).^42,43^ Histomorphological and morphometric analyses of the organs were performed. The methodology of morphometric analysis, detailed in SI Fig. S3 to S10, was carried out using Fiji Image J software.^44,45^

### Biochemical studies

#### Enzyme-linked immunosorbent assay

Commercially available FSH (Monobind Inc. USA), LH (Monobind Inc. USA), and T (PerkinElmer Inc. USA) hormonal immunoassay kits were purchased. Hormonal levels in blood plasma were quantified according to the kit specific protocols and the absorbance was recorded on UV-spectrophotometer (680xR, Bio-Rad, Tokyo, Japan).

### Antioxidant profiling and oxidative stress biomarkers

#### Homogenate preparation

17% W/V testicular tissue was homogenized in 100 mM Tris–HCl buffer (0.16 M KCl, pH = 7.5) at 14 500 rpm for 5 min (Heidolph-D91126 silent crusher). The homogenate was centrifuged (Scilogex, D3024) at 15 000 rpm and 4 °C for 90 s, and the supernatant was used for biochemical analysis.

#### Catalase (CAT)

The CAT concentration was assessed following the established protocols^46,47^ using a reaction mixture 200 μL phosphate buffer (PB) (50 mM, pH 7.6), 20 μL H_2_O_2_ (30 mM), and 100 μL of the homogenate. The absorbance was recorded at 240 nm (Thermoscientific spectrophotometer UV-3100PC) and concentration was estimated using an extinction coefficient of 43.6 M^−1^ cm^−1^.

#### Glutathione peroxidase (POD)

The assay mixture consist of 8 mM NADPH, 0.15 M glutathione, glutathione reductase, 2 mM H_2_O_2,_ and the homogenate, followed by incubation at 37 °C. The absorbance recorded at 340 nm and POD concentration was measured using an extinction coefficient (6.3 M^−1^ cm^−1^).^48,49^

#### Glutathione S transferase (GST)

The GST absorbance in the reaction mixture was measured following the protocols.^50,51^ The GST concentration was calculated using an extinction coefficient (9.6 mM^−1^ cm^−1^).

#### Guaiacol peroxidase (GPx)

The GPx concentration was determined by mixing homogenate with 0.1 M PB (pH 7.2), a 12.3 mM H2O2 solution, and 20 mM guaiacol, following slight modifications to the established protocols. The absorbance was recorded at 436 nm, and the GPx concentration was assessed using an extinction coefficient of 25 mM^−1^ cm^−1.^^49,52,53^

#### Glutathione reductase (GSR)

The GSR concentration was measured following the Carlberg & Mannervik protocol. The homogenate was incubated with 0.1 M PB (pH 7.0), 0.1 mM NADPH, 0.5 M EDTA, and 1 mM oxidized glutathione. The GSR concentration was quantified spectrophotometrically.^54^

#### Thiobarbituric acid reactive substance (TBARS) assay

Lipid peroxidation was quantified following Iqbal and Wright with little modifications.^55,56^ The homogenate was incubated with 0.58 mL of PB (0.1 M, pH 7.4), ascorbic acid (0.1 M), and 0.02 mL of FeCl_3_ (0.1 M) at 37 °C for 1 h. The reaction was stopped with 10% trichloroacetic acid, followed by the addition of 0.67% TBARS and then boiled for 30 min. Malondialdehyde (MDA) levels were assessed at 532 nm using an extinction coefficient of 1.56 × 10^6^ M^−1^ cm^−1.^^57^

#### Total reactive oxygen species (ROS)

The ROS concentration was estimated at 505 nm using the previously established protocols.^58,59^ A standard regression curve was generated using H_2_O_2_, and ROS levels were calculated from the equation *Y* = 0.0169*X* + 0.0983.

### Semen analysis

#### Sperm count

10 μL of semen from the cauda epididymis was diluted with normal saline (1 : 300), loaded onto an improved Neubauer chamber (0.1 mm^2^). The spermatozoa were counted in five large squares at 400× magnification (Olympus CX-41).^60–62^ The formula for spermatozoa concentration is given below:Concentration per mL = (dilution factor) (count in 5 squares) (0.05 × 10^6^)

#### Sperm viability

10 μL of diluted semen was poured onto a glass slide and mixed with 3 μL of 1% eosin solution and 2 μL of 10% nigrosin solution, air dried, and observed under a 40× objective lens; The viability is expressed as a percentage of live and dead spermatozoa.^63^

#### Sperm morphology

The spermatozoa smears were examined under the light microscope at 40× objective to record head, midpiece, and tail defects; spermatozoa were classified as abnormal and normal following minor modifications to the established protocols^64,65^

#### Sperm motility

10 μL of the diluted semen sample was poured onto a pre-warmed glass slide, coverslipped and examined at 100× magnification. Sperms were categorized as motile or non-motile.^66^

#### Statistical analysis

The results of the computed data were statistically interpreted using a one-way analysis of variance (ANOVA) performed with GraphPad Prism version 9.5.1 software. *Post hoc* Tukey's test was performed for multiple comparisons between various groups. All findings are expressed as mean ± SEM with a significance threshold of *P* < 0.05.

## Results

### Phytochemical analysis of LP

The LP phytochemical profile reveals the presence of bioactive compounds in aqueous, methanolic, and ethanolic extracts, as presented in Fig. S11 and Table 1. In the aqueous extract, the presence of tannins, saponins, flavonoids, steroids, polyphenols, alkaloids, anthraquinones, and coumarins, which act as natural reducing and stabilizing agents during the phyto-mechanochemical synthesis of LP@AgNPs, is consistent with the previous reports. Beyond their role in NPs formation, these phytoconstituents are well recognized for their fertility improvement and antioxidant properties.^67–69^ Collectively, these findings provide a mechanistic basis not only for the synthesis of NPs but also for their potential therapeutic effects.

**Table 1:** Phytochemical composition of aqueous, methanolic, and ethanolic extracts of LP^a^

Biomolecule	Aqueous extract	Methanolic extract	Ethanolic extract
Tannins	+	+	+
Saponins	+	+	+
Flavonoids	+	+	+
Steroids	+	+	+
Alkaloids	+	+	+
Polyphenols	+	+	+
Anthraquinone	+	+	+
Cardiac glycosides	−	+	+
Coumarins	+	+	+

a Keys: “+” = presence of a compound “−” = absence of a compound.

### Characterization of LP@AgNPs

#### UV-visible spectroscopy

**Fig. 2:**
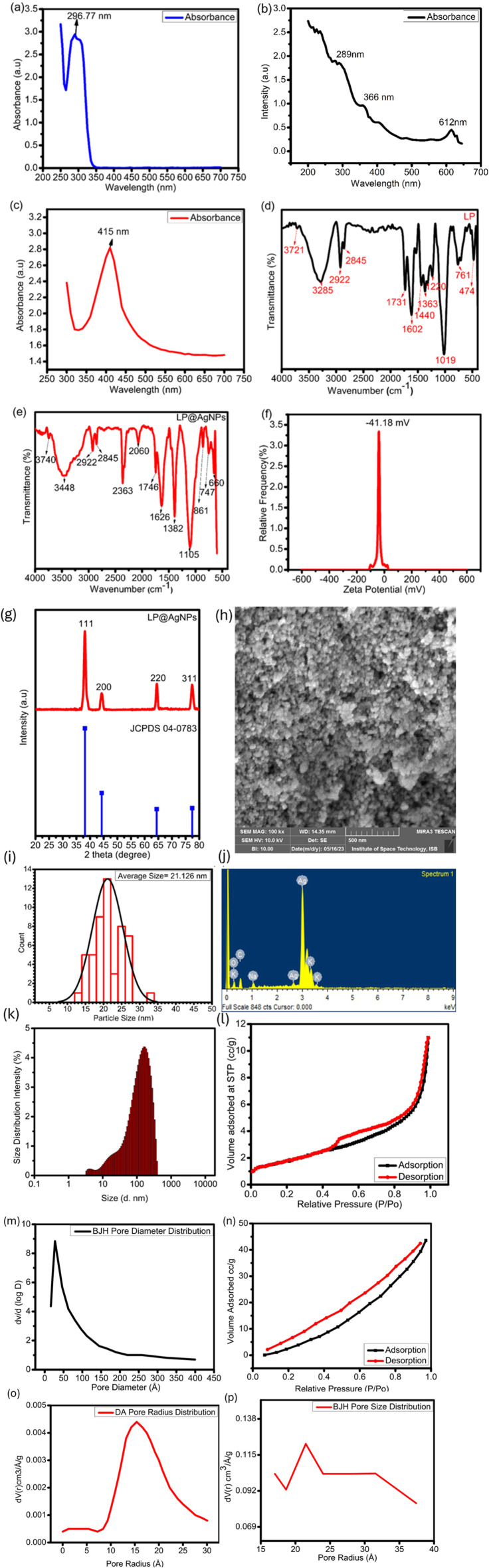
Characterization of LP@AgNPs, UV-visible spectra (a) AgNO_3_, (b) LP, and (c) LP@AgNPs, FTIR spectra of (d) LP, (e) LP@AgNPs, (f) zeta potential analysis of LP@AgNPs, (g) the PXRD spectrum demonstrating FCC pattern of the NPs (h) the SEM micrograph of LP@AgNPs crystals, (i) Histogram (j) the EDX spectrum showing the highest content of metallic Ag in LP@AgNPs, (k) the size distribution intensity of LP@AgNPs through dynamic light scattering (l) N_2_ adsorption–desorption isotherms of LP. (m) BJH pore size distribution of LP, (n) N_2_ adsorption–desorption isotherms of LP@AgNPs, (o) DA size distribution of LP@AgNPs, and (p) BJH pore size distribution of the NPs.

Ball milling of AgNO_3_ with an aqueous extract of the LP produces LP@AgNPs. The formation of the NPs was confirmed through UV-spectroscopy (Fig. 2(a–c)). The AgNO_3_ solution displayed a maximum absorbance peak at 296.77 nm. The UV-spectrum of the LP has shown three distinct peaks at 289 nm, 366 nm, and 612 nm. These peaks correspond to the phytochemicals present in the plant extract. The surface plasmon resonance at 415 nm indicates the formation of the LP@AgNPs. It also shows the ability of the phytochemicals present in the LP to reduce Ag from the Ag^+1^ state to the Ag^0^ state. Several factors, including initial concentration of AgNO_3_, the concentration of the extract, stirring time, and temperature, affected the yield of the NPs. The LP@AgNPs remained highly stable over a period of one year and they were stored in standard laboratory conditions at room temperature. The UV-Vis spectra captured after one year is shown in Fig.S12(a).

#### Fourier transform infrared spectroscopy

The FTIR spectrum of the LP demonstrated peaks at wavenumber: 3285 cm^−1^, 2922 cm^−1^, 2845 cm^−1^, 1731 cm^−1^, 1602 cm^−1^, 1440 cm^−1^, 1363 cm^−1^, 1325 cm^−1^, 1220 cm^−1^, 1019 cm^−1^, 761 cm^−1^, 474 cm^−1^. Contrarily, the LP@AgNPs demonstrated peaks at 3740 cm^−1^, 3448 cm^−1,^ 2922 cm^−1^, 2845 cm^−1^, 2363 cm^−1^, 2060 cm^−1^, 1746 cm^−1^, 1626 cm^−1^, 1382 cm^−1^, 1105 cm^−1^, 861 cm^−1^, 747 cm^−1^, and 660 cm^−1^ wavenumbers. The characteristic peaks at 3740 cm^−1^ and 3448 cm^−1^ represent free hydroxyl group and –OH stretching with intermolecular bounded forces, respectively. The peaks at 2922 cm^−1^ and 2845 cm^−1^ represent C–H bond and –CH_3_ respectively. A sharp peak 2363 cm^−1^ confirms the carboxylic acid group, indicating flavonoids in the LP extract. A peak at 1746 cm^−1^ corresponds to the ester group, and a peak at 1626 cm^−1^ represents the bio-fabrication of C=C on Ag. The sharp peak at 1382 cm^−1^ represents the C–O functional group, and the 1105 cm^−1^ peak identifies the alkyl amine group. A minor groove at 861 cm^−1^ represents the presence of aromatic compounds. So, the chief functional groups involved in the bio-reduction of Ag are carbonyl, hydroxyl, carboxylic, and aromatic (Fig. 2(d–e)).

#### Powder X-ray diffraction

In the PXRD pattern, four peaks were observed at 2*θ* values of 38, 44, 64, and 77° in correspondence with (111), (200), (220), and (311) planes respectively (Fig. 2(g)). The peaks corresponding to these planes indicate the FCC structure of the NPs (reference: Joint Committee of Powder Diffraction Standard card no (JPCDS-04-0783)).^70^ The crystallite size of the LP@AgNPs is 11.388 nm as calculated by using Debye Scherrer equation in Table 2.

**Table 2:** Crystallite size determination of LP@AgNPs using Debye Scherrer equation

Peak	2*θ* (deg)	*λ* (A)	FWHM (deg)	FWHM (rad)	Cos *θ*	*β* cosθ	*D* = *kλ*/*β* cos *θ* (nm)	Average particle size (nm)
1st	38	1.5406	0.7822	0.01375	0.945	0.01118	12.40	11.388 nm
2nd	44	1.5406	1.0301	0.01797	0.926	0.01665	8.32	
3rd	64	1.5406	0.6982	0.01218	0.846	0.01030	13.46	
4th	77	1.5406	0.8972	0.01566	0.781	0.01222	11.35	

#### Scanning electron microscopy

The SEM analysis was carried out to explore the morphology of the LP@AgNPs. The SEM micrographs revealed the spherical shape the NPs, with no agglomeration observed between the particles. The NPs are highly stable and capped with the LP bioactive compounds. The average grain size of the LP@AgNPs is 21.126 nm (Fig. 2(h)).

#### Energy dispersive X-ray spectroscopy

The EDX analysis also confirms the formation of LP@AgNPs as the corresponding peaks of Ag appeared on the spectrum (Fig. 2(j)). Owing to surface plasmon resonance, the EDX analysis showed a strong signal at 3.1 keV in the Ag region. The oxygen peak might be due to air oxidation during the formation of the SEM grid. Carbon and oxygen moieties might also be attributed to phytochemicals as reported in the previous study.^71^ Potassium and sodium spectral signals were also present due to adsorption on the surface of the LP@AgNPs from the LP extract. The quantitative measure of the elemental composition is also given in SI Table S1.

#### Zeta potential

The negative potential (−41.18 mV) value of the LP@AgNPs indicates the presence of bio-organic components that function as capping agents (Fig. 2(f)). The ZP, a physical property of NPs, gives information about the electrical state of charged interfaces. A high magnitude of ZP, whether positive or negative, is an indicator of strong electrostatic repulsion between the particles, thereby preventing aggregation. Thus the observed value confirmed the excellent colloidal and electrostatic stability.^72^ The LP@AgNPs demonstrated stable zeta potential over a period of one year under standard laboratory conditions confirming their long term colloidal stability. The ZP recorded after one year are shown in Fig. S12(b).

#### Dynamic light scattering

The DLS analysis shows that the size of the LP@AgNPs is 103.71 d. nm (Fig. 2(k)). The PDI of the LP@AgNPs is 0.279, with a width of 85.82 d. nm. As studies have indicated, NPs with PDI values greater than 0.7 are polydisperse, and PDI values smaller than 0.7 are monodisperse.^73^ The PDI value of 0.279 indicates the monodisperse nature of the LP@AgNPs. There exist heterogeneity in the NPs size, may arise from solvent interactions, as discussed in the discussion section. The zeta sizer generated data is given in Fig. S13.

#### BET analysis

The LP and LP@AgNPs were also characterised using BET analysis (Fig. 2(l–p)). The specific surface area and pore volume were calculated using the N_2_ adsorption–desorption method. The LP exhibited a type III BET isotherm, whereas the LP@AgNPs fell in the type IV BET isotherm category. The specific surface areas, pore diameters, and pore volumes of the LP and LP@AgNPs were 6.647 m^2^ g^−1^ & 67.98 m^2^ g^−1^, 27.303 A & 21.4865 A, and 8.842 cm^3^ g^−1^ & 0.1221 cm^3^ g^−1^, respectively. These results suggest that the LP@AgNPs are less porous and denser, having numerous tiny pores rather than a few large pores, compared to the LP.

#### Reproducibility of the phyto-mechanochemical method

The reproducibility of the phyto-mechanochemical method was confirmed by UV-visible spectroscopy across three independent batches, all of which consistently displayed a characteristic AgNPs surface plasmon resonance (SPR) band at 415.33 ± 1.25 nm with a FWHM of 70.68 ± 2.72 nm (Fig. S12(c)). The coefficient of variation for peak absorbance is 4.04% and the area under the curve is 8.61%. The overlapping SPR bands demonstrated the robustness of the method, producing LP@AgNPs with consistent size and optical properties across independent batches. Furthermore, a preliminary scale-up trial with a tenfold increase in precursor loading resulted in NPs with comparable yield and size distribution, thereby validating the scalability of the approach. The phyto-mechanochemical strategy demonstrated higher yields, reduced energy requirements, and greater reproducibility compared to the conventional phyto-synthesis and sol–gel method, while achieving efficiencies close to those of chemical reduction but without the associated toxicity. This validates the technique as an effective, eco-friendly, and scalable approach for producing AgNPs. The comparison of the phyto-mechanochemical method with other methods is given in Table S4.

#### Limitations of the phyto-mechanochemical method

The phyto-mechanochemical approach for synthesizing silver nanoparticles (AgNPs) has several notable advantages, including being solvent-free, eco-friendly, and energy-efficient. However, it is important to recognize its limitations:

1. The phytochemical composition of plant materials can vary based on factors such as the part of the plant used, the harvesting season, and the geographical origin. These variations may affect the reproducibility of nanoparticle size, shape, and yield.

2. Fine-tuning particle morphology, such as size distribution, shape uniformity, and crystallinity, requires high expertise in phyto-mechanochemical synthesis compared to conventional chemical or physical methods.

3. The precise roles of bioactive plant metabolites as reducing, stabilizing, and capping agents during mechanochemical reactions are not yet fully understood, which limits the ability to optimize the process accurately.

4. The incorporation of organic residues from plant materials can sometimes interfere with the surface purity of nanoparticles, affecting their stability and biological interactions.

Despite these limitations, phyto-mechanochemical synthesis offers a sustainable and innovative route for producing AgNPs. Future research focused on standardization, mechanistic understanding, and optimization for larger scales will further enhance its applicability.

#### Acute toxicity

No morphological abnormalities or mortality were recorded during the 14 days at any of the tested doses, confirming the safety of LP@AgNPs up to 1000 mg kg^−1^ body weight. The histopathological findings of the tests, kidney, and liver are presented in Fig. S14. These findings are consistent with previous studies that have reported this dosage range to be safe.^74,75^

#### Physiological observations and weight parameters

No mortality was recorded during the experimental period. The animals were healthy, exhibited normal feeding behavior, and showed no visible signs of biological anomalies. Following euthanasia, open organ harvesting was performed, and the organs were weighed immediately. SI Table S2 presents the changes in body and organ weights after 28 days of continuous exposure to the dosage across various groups. The mean ± SEM values of all the parameters were recorded. There was no significant difference in body, testes, epididymis, prostate, seminal vesicle, liver, kidney, and heart weights between treatment and control groups. The lack of significant differences in the body and organ weights indicates that the treatments did not induce systemic toxicity or adversely affect the maintenance and development of these organs.

### Histology

#### Morphological changes in the testes

**Fig. 3:**
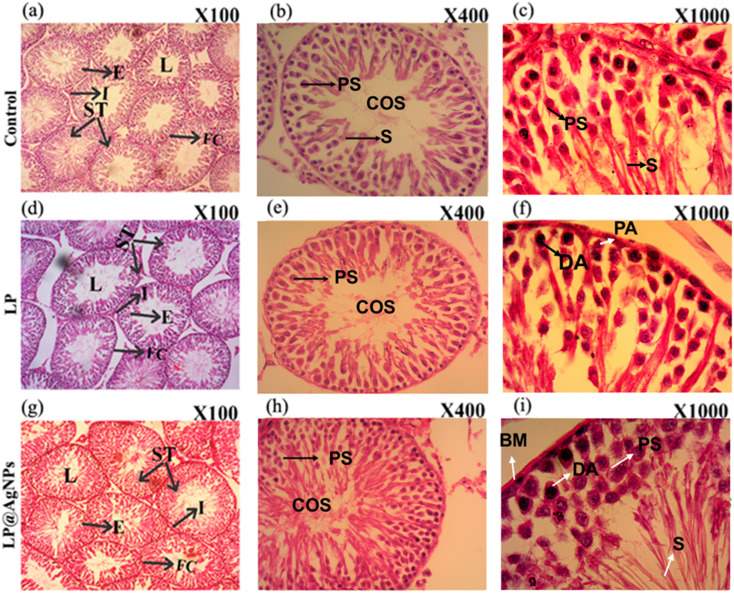
Representative cross-sectional photomicrographs of H & E stained STs at 100×, 400×, and 1000× in experimental groups of adult male Sprague Dawley rats. Photomicrographs (a–c) represent the control group, showing compactly arranged spermatogonia (STs), normal epithelium (E), and low levels of spermatids in lumens (L). Photomicrographs (d–f) correspond to the LP-treated group, depicting STs with normal E, intact leydig cells, and reduced spermatids. Photomicrographs (g–i) illustrate the NPs treated group, demonstrating a typical arrangement of STs, preserved E, and L densely packed with spermatozoa. Abbreviations: ST: seminiferous tubule, L: lumen, I: interstitial space, E: epithelium, FC: fibrous connective tissue, COS: clump of spermatozoa, PS: primary spermatocyte, S: spermatids, BM: basement membrane, DA: dark A-type spermatogonia, PA: pale A-type spermatogonia.

The testicular histopathological examination revealed an intact, typical seminiferous tubule (ST) structure in both the control and treated groups. All the tubules are covered with a thick layer of tunica albugenia. The STs are lined with germ cells organised in concentric layers displaying a complete spermatogenic process with all stages of the spermatogenic cycle. The intertubular spaces are composed of a loose connective tissue network, including flattened fibroblasts, capillaries, macrophages, mast cells, lymphatic vessels, and leydig cells. The arrangement of all the STs was similar in the control and drug-administered groups. The lumens of LP@AgNPs treated STs were filled with spermatozoa. However, in the case of the control and the LP-treated groups, they were empty, showing the lowest number of spermatozoa compared to that of the LP@AgNPs. The morphological changes in the testes are demonstrated in Fig. 3(a–i).

#### Histomorphometric changes in testes

**Fig. 4:**
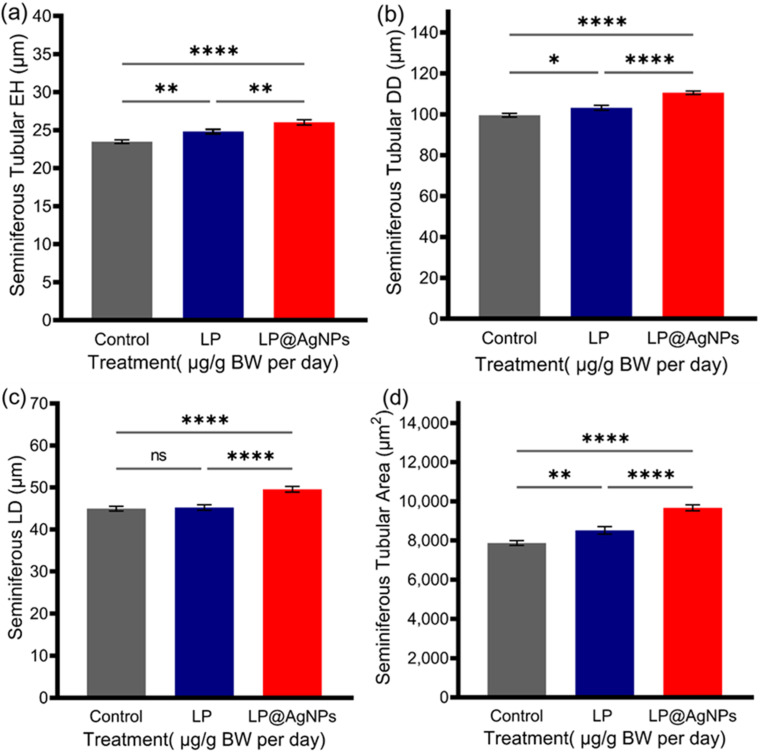
Histomorphometric analysis of ST parameters in response to drug treatments in adult male Sprague Dawley rats. Measurements include (a) seminiferous tubular EH, (b) DD, (c) LD, and (d) TA across the control, LP, and LP@AgNPs-treated groups. All the results in (a–d) are presented as mean ± SEM. *P* value ≤ 0.05 is represented as “*”; *P* value ≤ 0.01 is represented as “**”; *P* value ≤ 0.001 is represented as “***”; *P* value ≤ 0.0001 is represented as “****”, and “ns” represents non significance. The 95% confidence intervals and effect sizes (Cohen's d for pairwise comparison and partial *η*^2^ for ANOVA) are available in SI Table S3.

The histomorphometric analysis of STs (total number of tubules = 375; *n* = 125 per group with randomly chosen *n* = 25/animal) was carried out using Fiji Image J.^76^ Changes in ductal or tubular diameter (DD) were observed in all experimental groups. The DD increased significantly after the administration of the LP (*P* ≤ 0.05) and LP@AgNPs (*P* ≤ 0.0001) compared to the control. A significant increase in the LP@AgNPs (*P* ≤ 0.0001) administered group is also observed in the DD compared to the LP. The LP@AgNPs significantly increased lumen diameter (LD), epithelium height (EH), and tubular area (TA). Histomorphometric analysis of ST parameters in response to the drugs is given in Fig. 4(a–d).

#### Morphological changes in epididymis

**Fig. 5:**
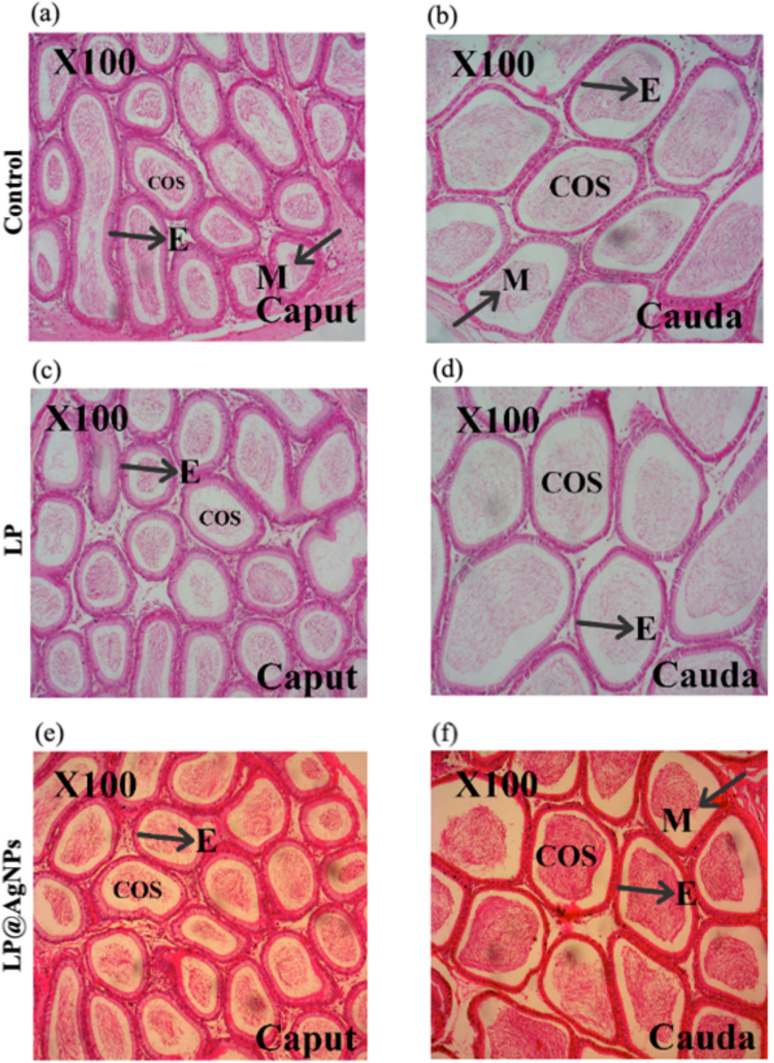
Representative cross-sectional photomicrographs of H & E stained caput and cauda epididymis at 100× in experimental groups of adult male Sprague Dawley rats. (a) The caput of the control group showing normal cellular artitecture, (b) the cauda of the control group at 100× magnification, showing typical E structure, COS, and M, (c) corresponds to the caput of the LP treated group showing reduced spermatozoa density, (d) represents cauda of the LP-treated group at 100× magnification, displaying altered E structure and reduced spermatozoa concentration. Photomicrographs (e and f) correspond to the LP@AgNPs-treated group at 100× magnification, highlighting preserved E integrity, M, and abundance in COS in the caput and cauda regions respectively. Abbreviations: E: epithelium, COS: clump of spermatozoa, M: musculature.

The histopathology of the control group presents the typical architecture of the epididymis cross-sections with regular tubules and pseudostratified columnar E with stereocilia. The tubular structure remained intact in all treated groups. The arrangements of the tubules in the treatment groups were similar to those in the control groups. A dense collection of spermatozoa was evident in the lumen of the LP@AgNPs-treated groups compared to the control and LP groups. Blood vessels (BVs) can be seen in the extra tubular space.The E was separated from the connective tissues through a basement membrane. Interstitial space did not change in treated groups in comparison to that of the control. All the morphological changes are illustrated in Fig. 5.

#### Histomorphometric changes in the epididymis

**Fig. 6:**
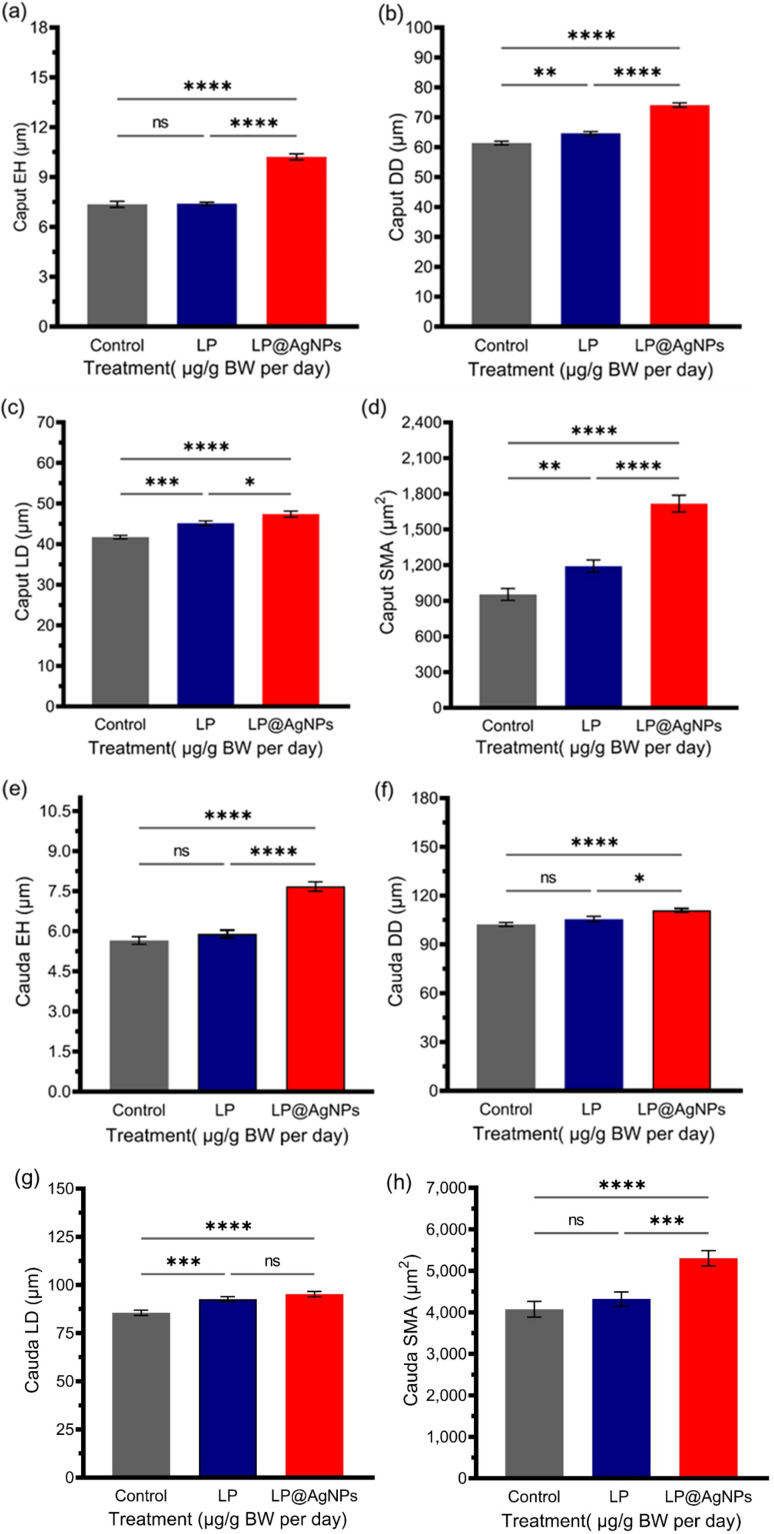
Histomorphometric analysis of the epididymis parameters in response to the drug treatments in adult male Sprague Dawley rats. The measurements include (a) caput EH, (b) caput DD, (c) caput LD, and (d) caput SMA, (e) cauda EH, (f) cauda DD (g) cauda LD, (h) cauda SMA. All the results in (a–h) are presented as mean ± SEM. *P* value ≤ 0.05 is represented as “*”; *P* value ≤ 0.01 is represented as “**”; *P* value ≤ 0.001 is represented as “***”; *P* value ≤ 0.0001 is represented as “****”, and “ns” represents non-significance. The 95% confidence intervals and effect sizes (Cohen's d for pairwise comparison and partial *η*^2^ for ANOVA) are available in SI Table S3.

The histomorphometric analysis of the epididymis presents significant alterations following drug treatment. Treatment with the LP alone did not induce substantial changes in the EH in the caput and cauda regions. However, the LP@AgNPs resulted in a marked increase in the EH compared to both the control (*P* ≤ 0.0001) and the LP@AgNPs treated group. Both the LP and LP@AgNPs significantly increased the DD in the caput region; only the LP@AgNPs elicited a pronounced increase in the DD in the cauda region compared to the control and the LP-administered groups. Furthermore, both treatments significantly enlarged the LD of the caput and cauda. The sperm mass area in these regions was also notably affected by the LP and LP@AgNPs, underscoring their substantial influence on epididymal morphology and function. All these changes are illustrated in Fig. 6.

#### Morphological changes in the ventral prostate

**Fig. 7:**
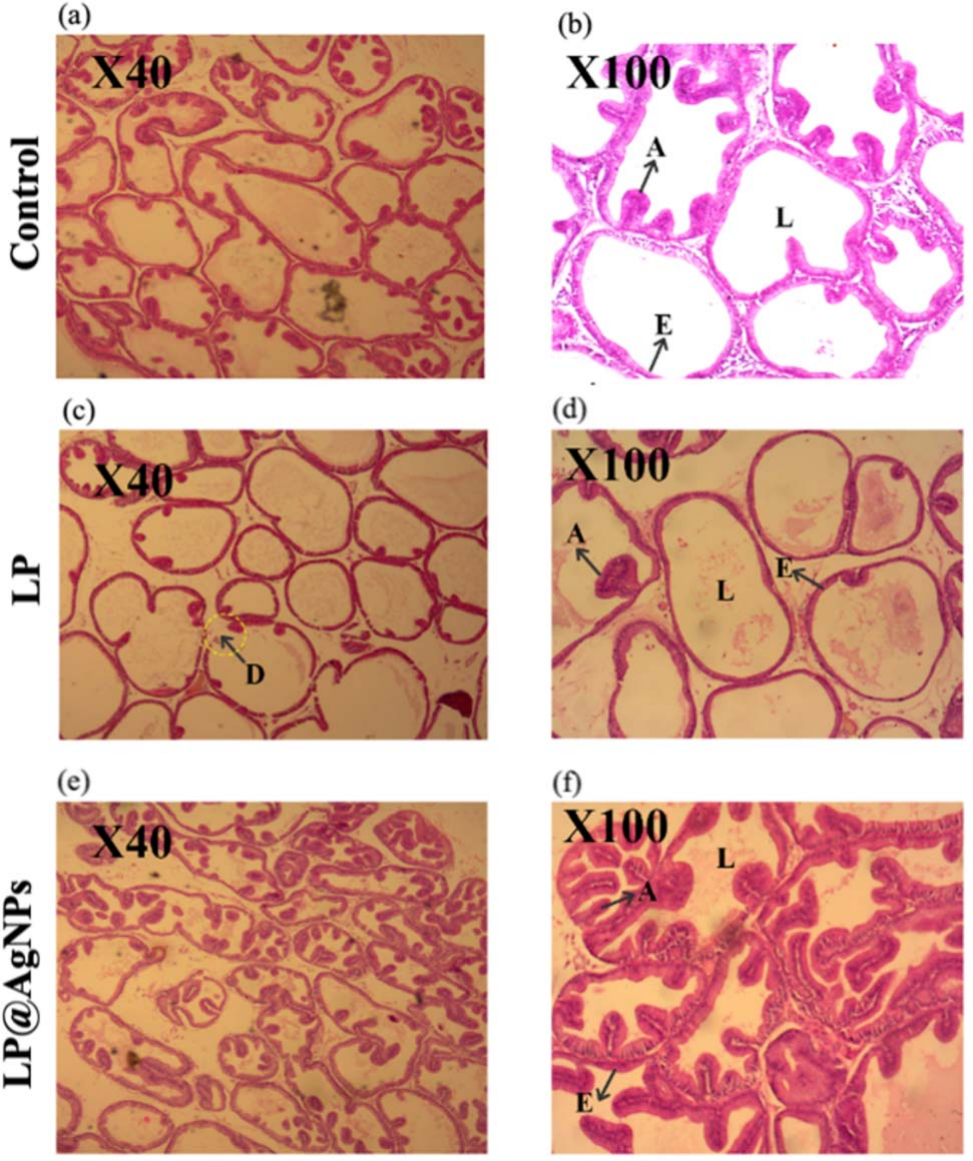
Representative cross-sectional photomicrographs of H & E-stained ventral prostate at 40× and 100× in experimental groups of adult male Sprague Dawley rats. Photomicrographs (a and b) represent the control group showing intact acini and normal prostate histology. Photomicrographs (c and d) correspond to the LP-treated group at 40× and 100× magnification, displaying E disruption, degenerated tubules, and altered luminal structure. Photomicrographs (e and f) display the effect of the LP@AgNPs treated group at 40× and 100× magnifications, highlighting preserved E integrity, acinar cell morphology, and L filled with secretions. Abbreviations: A: acini, D: degenerated tubules, E: epithelium, L: lumen.

In the control group, normal morphology with tightly packed prostatic acini of varying sizes and uniform shapes separated by a thin fibromuscular stroma was observed in histopathological cross-sections. The acini were bordered by a single layer of cuboidal cells with rounded vesicular nuclei, and some acini lumens were filled with acidophilic secretions. However, acinar dystrophy and tubular degeneration can be observed in the LP-administered group. The ventral lobe's acini revealed either the disappearance of the mucosal folds or that they were very few and shallow. Most of the tubules were intact, but they lacked any acini. The LP@AgNPs caused an increase in the number of acini in the tubules. The acinar E was a simple columnar with basophilic cytoplasm and basal nuclei on an intact basement membrane. All the acini contained eosinophilic secretory material, as shown in Fig. 7.

#### Morphometric changes in the ventral prostate

**Fig. 8:**
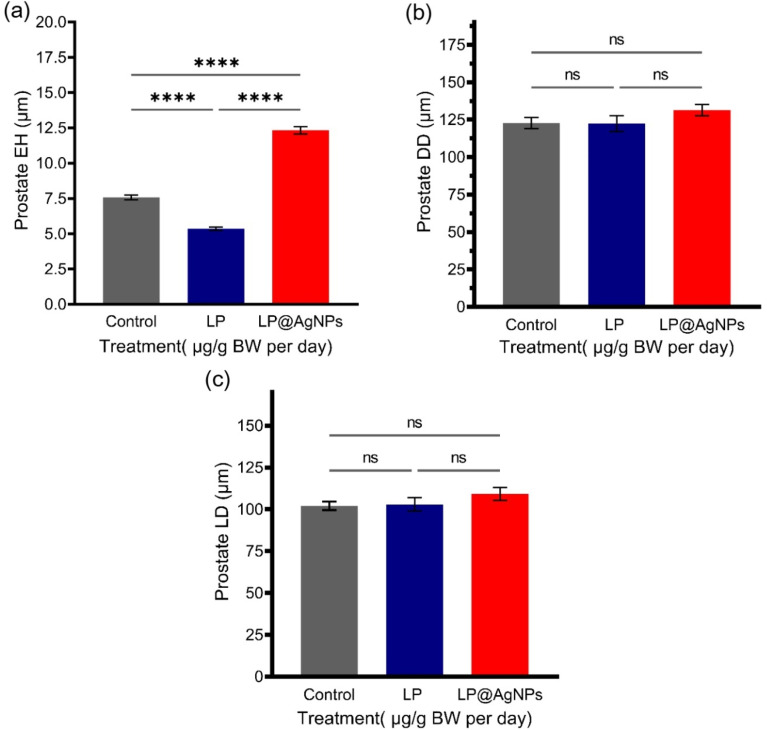
Histomorphometric analysis of the ventral prostate in response to drug treatments in adult male Sprague Dawley rats. Measurements include (a) EH, (b) DD, and (c) LD across the control, LP, and LP@AgNPs-treated groups. All the results in a–c are presented as mean ± SEM. *P* value ≤ 0.05 is represented as “*”; *P* value ≤ 0.01 is represented as “**”; *P* value ≤ 0.001 is represented as “***”; *P* value ≤ 0.0001 is represented as “****”, and “ns” represents non-significance. The 95% confidence intervals and effect sizes (Cohen's d for pairwise comparison and partial *η*^2^ for ANOVA) are available in supplementary Table S3.

The morphometric analysis of the prostate has displayed a significant impact of drug treatment on the prostate of Sprague Dawley rats. There was a significant (*P* value ≤ 0.0001) decrease in the EH of the prostate after treatment with the LP. However, the LP@AgNPs significantly increased the EH as compared to both the control (*P* value ≤ 0.0001) and LP-treated groups (*P* value ≤ 0.0001). The LP and LP@AgNPs have a non-significant impact on the DD and LD of the tubules, as shown in Fig. 8(a–c).

#### Seminal vesicles histology

**Fig. 9:**
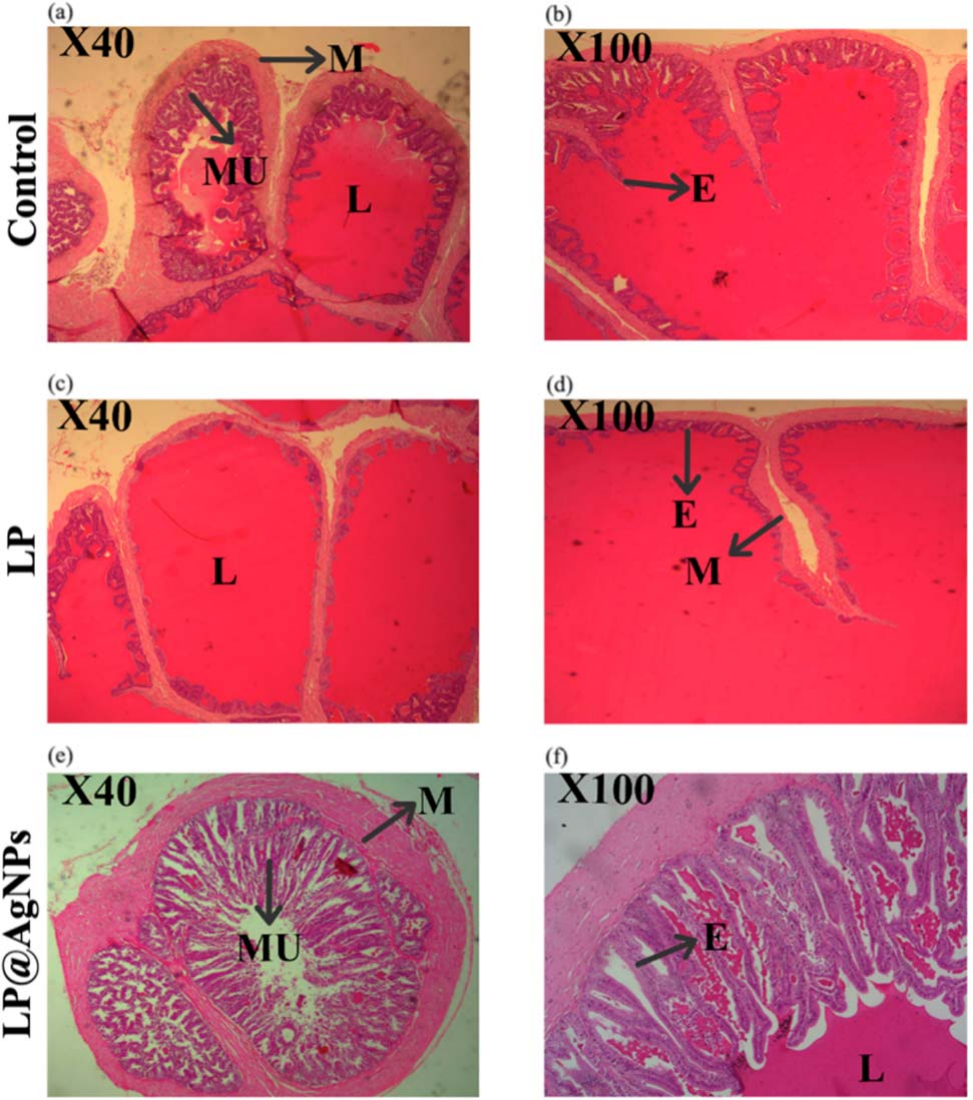
Representative cross-sectional photomicrographs of H & E-stained seminal vesicles at 100× in experimental groups of adult male Sprague Dawley rats. Photomicrographs (a, b) represent the control group at 40× and 100× magnifications, respectively, showing normal MU, M, and L. Photomicrographs (c, d) correspond to the LP-treated group at 40× and 100× magnification with evident E and M changes. Photomicrographs (e, f) display the LP@AgNPs treated group at 40× and 100× magnifications, highlighting intact MU, M, E, and L. Abbreviations: M: muscularis, MU: secretory mucosa, E: epithelium, L: lumen.

In a morphological analysis of the seminal vesicles, no histopathological changes were observed in any of the groups, including the control, LP, and LP@AgNPs-treated groups. The E of all groups was pseudo-stratified columnar. After treatment with the LP, there was a decrease in the secretory epithelium (desquamation of the epithelial lining with shortened folds) along with muscularis thickening in the respective group compared to that of the control. The mucosal degeneration can also be observed in the treated group. Conversely, the muscularis and epithelium thickened in the LP@AgNPs-treated group compared to the control and LP groups, demonstrating the efficacy of the LP@AgNPs, as shown in Fig. 9.

#### Systemic toxicity

**Fig. 10:**
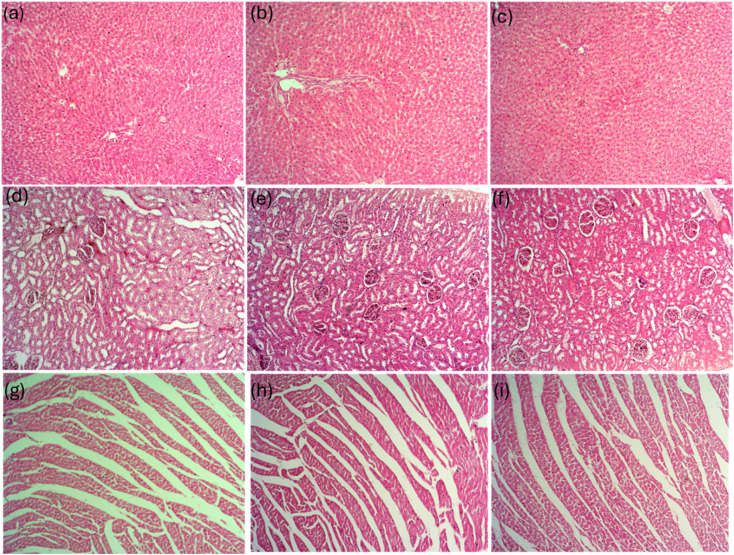
Representative cross-sectional photomicrographs of H & E-stained vital organs at 100× magnification in experimental groups of adult male Sprague Dawley rats. (a) Liver section of the control exhibited a typical arrangement of hepatocytes around the central vein, (b) Liver section of LP administered rats with a portal triad, (c) Liver sections of LP@AgNPs showed hepatocytes arranged in a typical pattern around the central vein, indicating the normal functionality. Kidney: (d) Control group depicted normal glomeruli and renal tubules, (e) LP-administered rats exhibited typical renal architecture, (f) LP@AgNPs treated rats demonstrated intact renal structures, with no signs of tubular degeneration, inflammatory infiltration, or necrosis. Heart: (g) Control group showed well-organized cardiac muscle fibers and blood capillaries, (h) LP-treated rats displayed standard cardiac tissue architecture, (i) LP@AgNPs-treated rats exhibited intact muscular fibers, with no signs of inflammation or cardiac injury, indicating maintained heart tissue integrity.

No visible signs of any hepatic, renal, or cardiac toxicity were observed following 28 days of administration of LP and LP@AgNPs. There was no evidence of NPs bioaccumulation, necrosis, or lesion formation in any of the examined organs. The hepatic histopathological architecture remained intact, with clearly defined central veins and portal triads. Hepatocytes exhibited normal morphology, with well-preserved nuclei, indicating that liver tissue structure and functions were not affected by the treatments Fig. 10(a–c). Similarly, the renal histopathological architecture remained typical in the treatment groups compared with the control. There were no signs of inflammatory infiltration, tubular degeneration, or tissue damage. The structure from the renal cortex to the renal medulla was well preserved, indicating that kidney architecture and function are standard (Fig. 10(d–f)). Furthermore, cardiac histopathology appeared normal, with well-organized cardiac muscle fibers, intercalated discs, and no evidence of inflammation or degeneration Fig. 10(g–i).

#### Hormonal profile

**Fig. 11:**
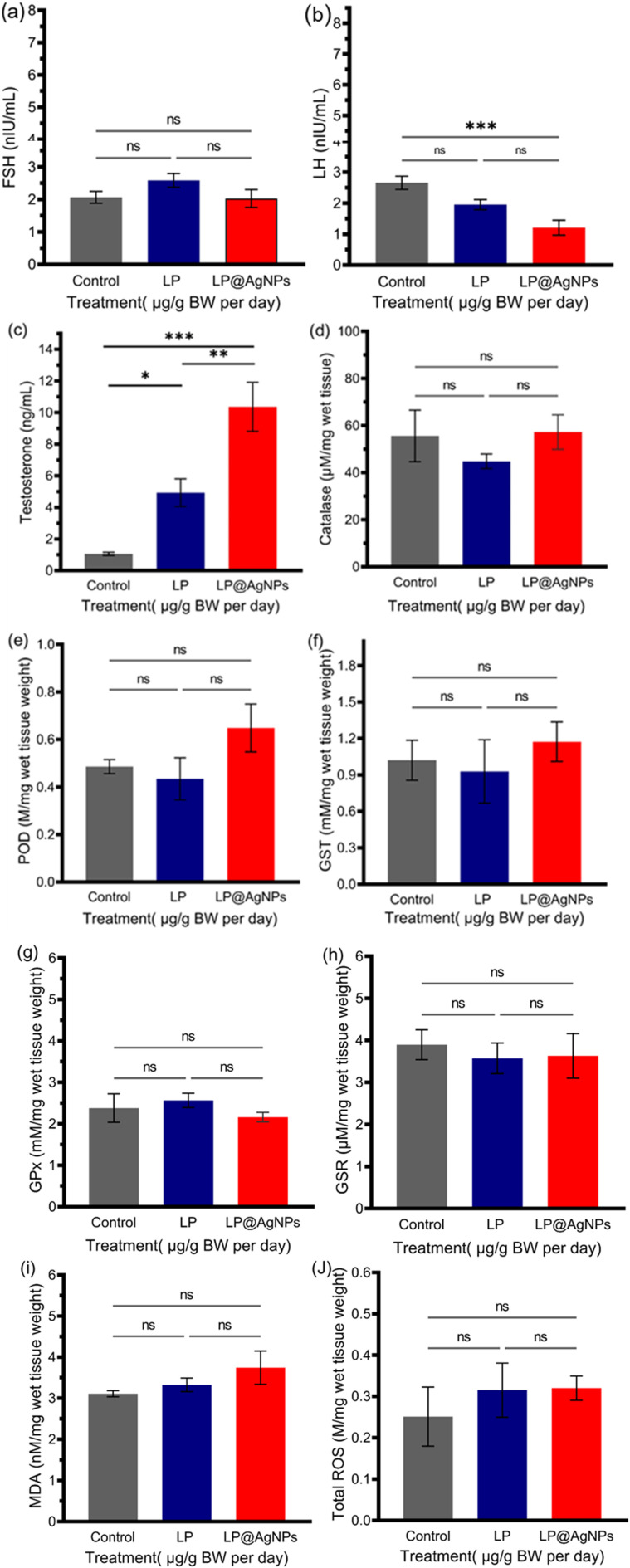
Effects of drug treatments on the biochemical profile of blood serum and tissue homogenates of adult male Sprague Dawley rats. (a) No significant change in concentration of FSH after the LP and LP@AgNPs administration, (b) The LP@AgNPs reduced the serum LH concentration, (c) the NPs boosted the serum T levels, indicating healthy functioning of Leydig cells, (d) CAT levels remained statistically unchanged across all treatment groups, (e) no significant changes in POD activity are observed. However, the LP@AgNPs group showed a non-significant upward trend. (f) GST levels were not significantly affected by any of the treatments. (g) No significant difference in GPx activity was observed among the control, LP, and LP@AgNPs groups. (h) GSR activity remained statistically unchanged across all treatment groups. (i) MDA levels showed a non-significant change among the three groups. (j) Total ROS levels were not affected by either LP@AgNPs or LP treatments compared to the control. All the results in a-j are presented as mean ± SEM. *P* value ≤ 0.05 is represented as “*”; *P* value ≤ 0.01 is represented as “**”; *P* value ≤ 0.001 is represented as “***”; *P* value ≤ 0.0001 is represented as “****”, and “ns” represents non-significance. The 95% confidence intervals and effect sizes (Cohen's d for pairwise comparison and partial *η*^2^ for ANOVA) are available in SI Table S3.

The LP and LP@AgNPs significantly affected the concentrations of key reproductive hormones. FSH levels were non-significantly reduced in the LP@AgNPs compared to the control and LP groups. LH levels were significantly reduced (*P* = 0.001) in the NPs-treated group compared to the control. T concentration was also significantly (*P* = 0.0001) elevated in the NPs-administered group compared to the control. These findings suggest that both LP and LP@AgNPs effectively regulate hormonal levels through the negative feedback mechanism of the hypothalamic-pituitary-gonadal axis, with the LP@AgNPs administration having a more pronounced effect due to the synergistic properties of the NPs. The results are presented as bar charts in Fig. 11(a–c).

#### Antioxidants and oxidative stress biomarkers

Treatments with LP and LP@AgNPs did not significantly enhance the key testicular antioxidants, including CAT, POD, and GST in testicular tissue homogenate. The CAT activity exhibited a non-significant increase in the LP@AgNPs group, compared to both the control and LP groups. Similarly, POD and GST activities were higher in the NPs-treated group; however, these changes did not reach statistical significance (*P* > 0.05). The GPx and GSR levels remained comparable across all groups, with no significant variation. Lipid peroxidation was assessed *via* MDA levels, which were the highest in the LP@AgNPs, though the increase was non-significant. However, ROS levels were reduced compared to the control in both the LP and LP@AgNPs. These findings suggest that LP and LP@AgNPs have not disturbed the oxidants-antioxidant balance in testicular tissue homogenates. The results are shown in Fig. 11(d–j).

#### Semen analysis

**Fig. 12:**
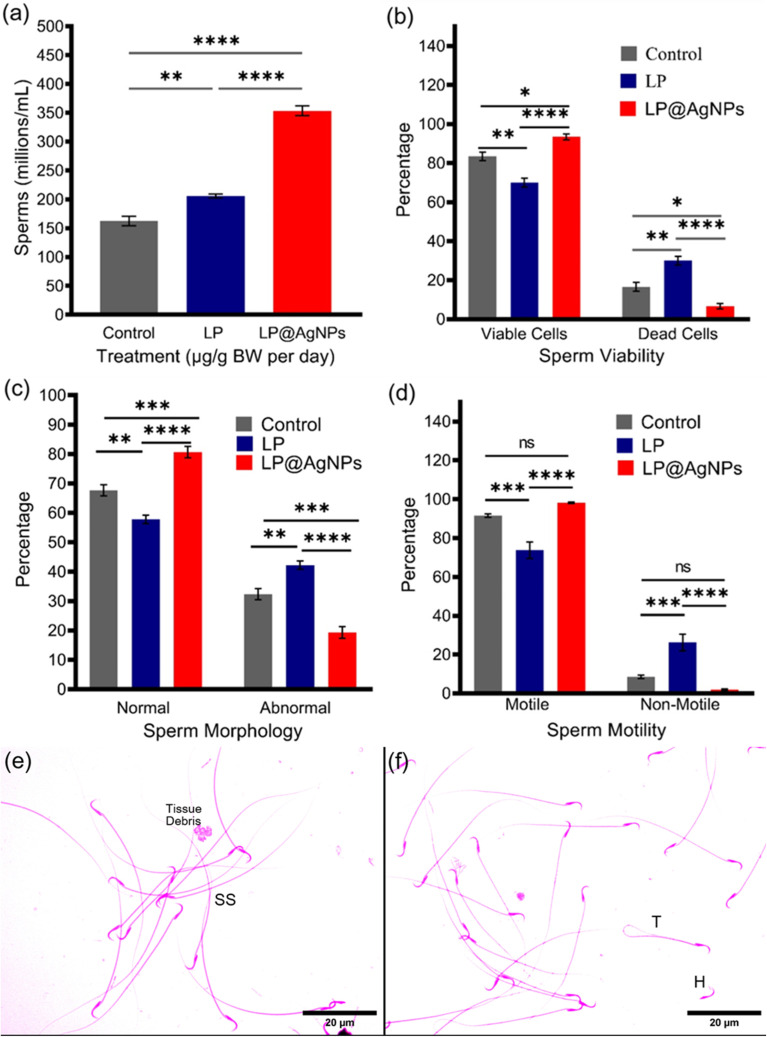
Drug-induced alterations in semen parameters of adult male Sprague Dawley rats. (a) Represents the significant increase in concentration of spermatozoa in millions per mL after the LP@AgNPs administration (b) the viability of the LP@AgNPs administered spermatozoa increased significantly after 28 days of exposure, (c) overall morphological abnormalities were reduced considerably in the NPs exposed group, (d) sperm motility did not differ significantly from the control group; however, a significant improvement was observed in comparison to the LP-treated group, (e) and (f) display morphological abnormalities in spermatozoa structure observed under experimental conditions. Highlighted anomalies include H: head without tail, T: bent tail, and SS: sickle-shaped spermatozoa. All the results in a–d are presented as mean ± SEM. *P* value ≤ 0.05 is represented as “*”; *P* value ≤ 0.01 is represented as “**”; *P* value ≤ 0.001 is represented as “***”; *P* value ≤ 0.0001 is represented as “****”, and “ns” represents non-significance. The 95% confidence intervals and effect sizes (Cohen's d for pairwise comparison and partial *η*^2^ for ANOVA) are available in SI Table S3.

All the results of semen analysis are presented in Fig. 12(a–f).

#### Sperm count

The epididymal sperm count was increased after treatment with the LP and LP@AgNPs in adult male Sprague Dawley rats. A significant improvement in sperm count was observed in the LP (*P* ≤ 0.01) and LP@AgNPs (*P* ≤ 0.0001) groups compared to the control group. The comparison of the LP and LP@AgNPs demonstrated that the latter was a more effective agent in significantly increasing the count (*P* ≤ 0.0001).

#### Sperm viability

After treatment with the NPs, a statistically significant increase in the percentage of live spermatozoa was observed compared to the control (*P* ≤ 0.05) and LP-treated group (*P* ≤ 0.0001). A significant reduction (*P* ≤ 0.01) in live spermatozoa was also detected in the LP-treated group compared to the control.

#### Sperm morphology

The sperm morphology was also influenced after the administration of the LP and LP@AgNPs. The spermatozoa were characterized into two categories: abnormal and normal. The percentage of abnormal spermatozoa was significantly the highest (*P* ≤ 0.01) in the LP-treated group compared to the control and the LP@AgNPs group. However, the NPs treatment improved the morphological parameters significantly.

#### Sperm motility

The spermatozoa were categorized into motile and non-motile groups. The treatment of the LP mainly impacted the motility of spermatozoa. The LP caused a significant reduction (*P* ≤ 0.001) in the percentage of motile spermatozoa compared to the control and LP@AgNPs. On the other hand, the LP@AgNPs had the highest percentage of motility, but this difference was non-significant compared to the control.

## Discussion

The global decline in sperm count and the increasing prevalence of male infertility, particularly across Asia, Europe, Africa, and North America, highlight a significant global health challenge.^9,77^ Conventional therapeutic approaches, including the aromatase inhibitor anastrozole, have shown limited efficacy and are associated with adverse effects such as testicular atrophy and histopathological alterations in reproductive tissues. Similarly, pharmaceutics, especially sertraline,^78^ fluoxetine,^79,80^ and pain medications,^81^ exacerbate male infertility by disrupting hormonal balance, reducing semen quality, and increasing sperm DNA damage. Clomiphene citrate, a widely used therapeutic agent in male reproductive disorders, is associated with serious side effects such as mood disorders, visual disturbance, and gynecomastia.^82^ Moreover, the high cost of ARTs and hormonal therapies in developing regions, such as Africa and South Asia, poses significant barriers.^83–86^ In this context, the LP@AgNPs emerge as a promising therapeutic alternative with the potential to alleviate the adverse effects of these factors. Their antioxidative and anti-inflammatory properties have shown efficacy in combating oxidative stress, protecting reproductive tissues, and restoring hormonal balance. The LP@AgNPs offer a new, cost-effective solution to enhance semen quality, support spermatogenesis, improve the hormonal profile, and address the multifaceted cause of male infertility.

LP, a cruciferous species, contains numerous bioactive compounds, including alkaloids, tannins, saponins, flavonoids, steroids, polyphenols, anthraquinones, cardiac glycosides, and coumarins. In addition to these compounds, it is rich in vitamin C and fiber.^87,88^ The current study proved that these bioactive compounds could potentially treat infertility-related problems in males with greater efficacy when they form LP@AgNPs in the animal system.

The UV-visible study confirmed the formation of the LP@AgNPs. The surface plasmon resonance band displayed at 415 nm confirmed the presence of the NPs, which aligns with previous studies.^89–91^ Surface plasmon resonance is a unique characteristic of metallic NPs that arises from oscillations of conduction band electrons.^92^ From the spectrum, the LP@AgNPs possess unique optical characteristics that are associated with their size and morphology. The UV-spectrum is crucial for determining the size, shape, and stability of NPs.

The FTIR results of LP align with previous studies,^88^ which confirmed the glucosinolates peak at 1022 cm^−1^. However, this characteristic peak cannot be seen in the LP@AgNPs. The PXRD results confirmed the presence of FCC crystals with a crystallite size of 12.775 nm, which aligns with the studies of.^70,93,94^ The PXRD pattern reveals no detectable impurity peaks, indicating the high purity of NPs. SEM images confirmed the formation of crystalline-shaped AgNPs with an average particle size of 21.126 nm, and the results correlate with the previous work.^89,95,96^

The EDX spectroscopy revealed the elemental composition of the LP@AgNPs. There was a strong signal of Ag at 3.1 keV, consistent with the previous literature.^97^ Other signals, such as potassium, sodium, carbon, and oxygen, represent organic moieties coated on the surface of the LP@AgNPs, as evidenced earlier.^98,99^ The potassium and sodium signals in the EDX of NPs may be due to LP's high potassium and sodium content.^100^

The ZP of the LP@AgNPs demonstrated a strong signal peak at −41.18 mV, representing their high stability. Anionic compounds are present on the surface of LP@AgNPs as capping agents, playing crucial role in stabilizing these NPs.^99,101,102^ Zeta sizer shows that the average diameter of the LP@AgNPs is 103.72 d. nm. The measured size of LP@AgNPs using a zeta sizer is more significant compared to that of SEM and aligns with previous literature.^72,103^

A noticeable discrepancy in the PXRD (11.388 nm) and DLS sizes (103.72 d. nm) was observed. The difference arises as the PXRD measures the crystallite size of the NPs, while the DLS reflects the hydrodynamic diameter. The hydrodynamic diameter includes the particle core, solvation shell, and adsorbed impurities. Factors such as particle concentration, surface charge, dispersion medium, organic coating, or swelling in a liquid can promote aggregation, leading to inflated DLS values.^104,105^ Thus, the heterogeneity and larger particle size in DLS are likely due to solvent effects rather than an actual increase in particle size.

The BET analysis revealed significant differences in the surface properties of the LP and LP@AgNPs, which may have implications for their ameliorative effects in the reproductive tract of rats. This study revealed that the LP exhibited a type III isotherm indicative of a non-porous or macroporous structure. At the same time, the LP@AgNPs displayed a type IV isotherm, suggesting the presence of mesopores. This distinction is crucial as mesoporous structures are known to have enhanced surface interactions, which could be beneficial for drug delivery systems and other biomedical applications where surface area plays a pivotal role in efficacy.^106^ The increased surface area for the LP@AgNPs provides more active sites, as reported in previous studies,^107,108^ creating more biological interactions that could enhance antimicrobial properties, reduce infections, and improve reproductive health outcomes. The LP has higher pore volume; the density and distribution of pores in the NPs are more favorable for applications requiring a high surface area-to-volume ratio. This is important in reproductive health, where the controlled release of antimicrobial agents can prevent infections that adversely affect reproductive health.^109,110^ The pore diameter measurements indicate that the LP@AgNPs have numerous smaller pores, which means they have more active sites available for chemical reactions and interaction with microorganisms, which is beneficial in antibacterial applications. The efficacy of the LP@AgNPs in inhibiting bacterial growth can lead to reduced infection rates, thereby enhancing reproductive health outcomes.^111,112^

In our experiment, neither the LP nor LP@AgNPs altered the weight of the body and reproductive organs after 28 days. Previous experiments have shown that red maca reduces prostate weight without affecting the testes, epididymis, seminal vesicles, liver, kidney, and heart.^88,113,114^ In our studies, there was no significant prostate weight loss in comparison to that of the control after the LP and LP@AgNPs treatment.

The results also demonstrated an increase in seminiferous tubular diameter, lumen diameter, epithelium height, and tubular area after applying LP@AgNPs. An increase in these parameters indicates enhanced spermatogenesis.^115^ Moreover, the density of spermatozoa per unit volume in the lumen of the tubules further supports the effectiveness of the NPs as a potential agent for treating infertility. Previous studies have suggested that NPs can cross the blood-testes barrier (BTB), with particles up to 50 nm capable of penetration, and smaller particles exhibiting more pronounced impacts.^116–119^ The LP@AgNPs might have crossed the BTB due to their small size, potentially enhancing the delivery and biological efficacy of the phyto-compounds on their surface in testicular tissues, thereby exerting more potent effects.

The bioactive compounds of the LP, whether in the LP powder or as capping agents for Ag, have been shown to influence the morphology of STs, particularly in terms of DD and LD. The current research suggests that the LP and LP@AgNPs enhance spermatogenesis, which is associated with an increase in the diameter of the STs. This effect is likely due to the stimulation of sertoli cells and the proliferation of germ cells, which contributes to the overall growth of STs.^120,121^ The increase in STs diameter is often correlated with the health and activity of spermatogenic cells and the hormonal environment, mainly testosterone, inhibin, and follicle-stimulating hormone levels, which are essential for spermatogenesis.^121–123^

In contrast, the prostate ductal diameter did not change significantly after applying the LP and LP@AgNPs. The morphology and functions of the prostate are regulated differently from those of testes and epididymis. The ductal structures in the prostate are less responsive to the same hormonal stimuli that affect the STs, which may explain the lack of change in ductal diameter despite treatment.^123,124^ Androgens primarily influence the prostate ductal system. However, the specific pathways and cellular responses differ from those in the testicular and epididymal tissues.^125,126^ However, the increase in DD in the epididymis can be attributed to sperm maturation and functional adaptations of the epididymal epithelium, which is designed to support sperm storage and maturation.^127^ The variation in LDs across the testes, epididymis, and prostate is associated with alterations in cellular morphology and function, to maximize reproductive functions affecting fluid dynamics within the tubules of reproductive organs.^128^

**Fig. 13:**
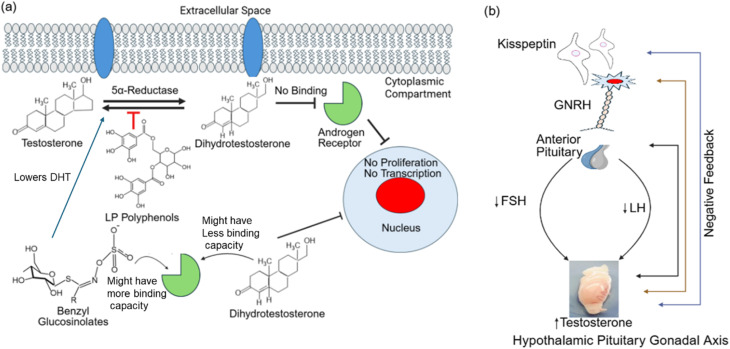
Possible mechanistic insights (a) prostate epithelium height reduction in response to LP treatment (b) hypothalamic-pituitary-gonadal axis modulation in response to LP@AgNPs.

The T administration in castrated rats suggested that the EH of the prostate is androgen-dependent.^129^ The LP is involved in treating benign prostatic hyperplasia and causes a reduction in prostate EH, which aligns with our results.^130^ The LP contains higher levels of glucosinolates, which are aromatic and benzyl glucosinolates.^131^ These glucosinolates act as antagonists for androgenic receptors present in the prostate due to their competitive binding capacity with androgens.^88^ A characteristic peak corresponding to 1019 cm^−1^ in the LP confirmed the presence of glucosinolates,^113^ whereas no such glucosinolates were observed on the LP@AgNPs. LP-based glucosinolates are associated with the reduction in EH of the prostate, and the mechanistic insight is shown in Fig. 13(a). Zou *et al.* reported that glucosinolates from *L. meyeni* reduced dihydrotestosterone (DHT) concentration, which may contribute to the reduction of prostate epithelium. A similar mechanism is also observed with finasteride, a 5-alpha reductase inhibitor used in the management of benign prostatic hyperplasia, where suppression of DHT synthesis in the prostate leads to diminished epithelial growth.^132,133^ Furthermore, glucosinolates may influence androgen receptor (AR) signaling by modulating pathways involved in receptor activation. In particular, isothiocyanates such as sulforaphane have been shown to affect histone acetyltransferase activity, thereby changing AR receptor functions and contributing to the disruption of proliferative signaling pathways.^134^ Collectively, these findings support the possibility that glucosinolates contribute to prostate epithelial reduction through a dual mechanism involving both decreased DHT and direct modulation of AR signaling. Further research is required to elucidate these pathways.

In addition to glucosinolates, polyphenols may also impact the action of T on androgen receptors as they inhibit 5-α reductase activity.^135^ The increased estrogenic hormone (EH) after administration of the LP@AgNPs may be linked to the modulation of estrogenic receptor ERβ^136^ and androgenic receptors^137^ in the prostate and their subsequent pathways. As EH growth depends on androgens,^138^ higher levels of T in the NPs-treated group may be a factor in improving EH in the seminiferous tubules, epididymis, prostate, and seminal vesicles. The NPs consistently exhibited unique properties, including antibacterial and anticancerous effects.^139,140^ The antibacterial potential of the LP@AgNPs could be another reason for the increase in EH of the testes, epididymis, prostate, and seminal vesicles, which needs further investigation.

In the present study, treatment with LP and LP@AgNPs significantly modulated reproductive hormones, including FSH, LH, and T levels. The FSH levels remained significantly unchanged, LH levels were significantly reduced, and T levels were markedly increased in the LP@AgNPs group compared to the LP and control groups. These findings suggest that the NPs influence the HPG axis *via* negative feedback driven by increased testosterone production, as shown in Fig. 13(b). The proposed mechanism is well established in reproductive physiology, whereby high concentrations of testicular steroids exert negative feedback on the hypothalamus or *vice versa*. Specifically, they act *via* kisspeptin neurons, GnRH neurons, and directly on pituitary gonadotrophs to support the HPG axis.^141,142^ The increased testosterone concentration may result from the phytochemical constituents of the LP, particularly saponins, steroids, or flavonoids, which are known to stimulate leydig cell functions and androgen synthesis.^143,144^ Additionally, the integration of LP bioactive compounds into AgNPs enhanced the efficacy of the bioactive compounds and augmented their biological activity. Previous studies have demonstrated that LP@AgNPs may enhance plasma testosterone levels by influencing the HPG axis (GnRH, FSH, and LH levels).^145,146^ The LP@AgNPs offer a promising approach to enhancing reproductive hormone regulation, particularly FSH and LH, by combining the LP adaptogenic and phytoestrogen effects, as discussed in the previous studies of the wild red maca plant.^147–149^ The LP@AgNPs can enhance the bioavailability and cellular uptake of bioactive compounds of the LP potentially leading to a more sustained impact on the HPG axis.^150^ Additionally, LP@AgNPs may synergistically amplify LP effects on T production, creating feedback that influences FSH and LH levels, making this a novel strategy for reproductive health.^151^

Antioxidants: the findings suggest that after the LP and LP@AgNPs treatment, a statistically significant antioxidant profile was not produced compared to the control. Different maca-based bioactive components play active roles in enhancing testicular antioxidant capacity. It has been reported that the LP-based *N*-benzylinoleamide and *N*-benzyloleamide lower MDA levels in brain and liver tissues, effectively protecting against oxidative damage.^152^ The LP-based macaridines, β-carboline alkaloids, and macamides demonstrate antioxidant potential *in vitro*.^153^ Polyphenols, polysaccharides, and alkamides might play a significant role in maintaining the antioxidant levels of treatment groups in homeostatic conditions, as reported in previous studies.^154–156^ The modulation of the antioxidant defense mechanism highlighted the potential benefits of LP and LP@AgNPs. It provided avenues for future research on developing therapeutic strategies for oxidative stress-related disorders.

The LP@AgNPs improved semen parameters compared to the LP-treated groups, demonstrating their effectiveness as an agent in the reproductive tract of rats. The LP@AgNPs improved the sperm count due to the presence of biologically active functional groups fabricated on the surface of metallic Ag. Earlier studies have shown that bioactive components, such as alkaloids,^157^ phenols,^158^ and flavonoids^159^ have been associated with improved sperm count. The phytochemical analysis of the LP, FTIR of the LP, and FTIR of the LP@AgNPs indicated the presence of the above-mentioned bioactive components in dosages of different drugs. However, it is challenging to identify which specific component enhances sperm count in the LP and LP@AgNPs treatment groups. Moreover, it has been documented that the LP biocomponents enhance sperm production by affecting the stages of mitosis, specifically the initial stages IX–XIV.^160^ The current result of the increment in sperm count after the application of the LP is in concordance with previous studies,^161^ which reported that red maca increases sperm count in the vas deferens. This finding contrasts with,^162^ where no effect was documented. The LP@AgNPs, a new agent formed from the bio-reduction of silver, showed greater efficacy in increasing sperm count compared to the administration of LP.

In the present study, the administration of LP resulted in decreased sperm viability and motility, likely due to its cytotoxic effects on the prostate and seminal vesicles. Notably, the absence of glucosinolates in LP@AgNPs appears to be associated with reduced sperm toxicity. The LP's glucosinolates have been reported to decrease the epithelial surface area of the prostate and seminal vesicles, thereby compromising their secretory functions, as discussed earlier. The prostate epithelium typically contributes zinc, citrate, and proteins, whereas the seminal vesicles secrete prostaglandins, fructose, and proteins essential for sperm vitality. The reduction in the epithelial surface consequently diminishes the glandular secretion, leading to several downstream effects. The altered composition of seminal plasma compromises antioxidant defenses, resulting in increased susceptibility to oxidative stress and structural damage. The reduced fructose availability limits adenosine triphosphate (ATP) generation, while the diminished prostaglandin levels further compromise the spermatozoa's progressive motility. The disruption of pH and ionic balance also creates a suboptimal environment for the spermatozoa's functions. Moreover, prostatic zinc provides bacterial protection in seminal plasma; its reduction may increase microbial susceptibility and lower sperm viability.^163–166^ In contrast, the LP@AgNPs appeared to exert a reverse effect, enhancing the epithelial surface of both glands and increasing their secretory functions. This increase in epithelial activity was reflected in improved sperm viability and motility relative to LP-treated and control groups.

Sperm morphology is adversely affected by supplementation with the LP suspension, likely due to its impact on prostatic and seminal vesicle secretions. Impaired secretory function limits the availability of essential nutrients, thereby compromising the structural integrity of spermatozoa. The reduced antioxidant capacity and zinc levels in seminal plasma may further increase oxidative stress and destabilize chromatin, leading to membrane disruption and DNA damage. A positive correlation exists between seminal plasma zinc content and normal sperm morphology, supporting the critical role of zinc in maintaining spermatozoa structural integrity. Moreover, seminal zinc concentration has been associated with pathological conditions like asthenozoospermia, azoospermia, oligozoospermia, and oligoasthenozoospermia.^167–169^ Flores *et al.* also reported that the abnormal prostatic secretions are associated with impaired spermatozoa development and morphology.^170^

Additionally, the presence of glucosinolates and their metabolites in LP may aggravate spermatozoa abnormalities by promoting intracellular ROS accumulation and mitochondrial dysfunction.^171^ Consistent with this, higher glucosinolate levels have been associated with anti-reproductive potential as discussed in the previous study.^172^ In contrast, the LP@AgNPs enhanced the secretory activity of the prostate and seminal vesicle, which may have improved nutrient availability, zinc secretion, and antioxidant support, thereby alleviating spermatozoa morphological defects. Furthermore, the male reproductive tract harbors a diverse microbiota,^173^ which can directly impair spermatozoa function by activating proinflammatory pathways and inducing oxidative stress, leading to poor semen quality.^174^ Elevated microbial load is commonly observed in pathologies such as epididymitis and prostatitis, where low semen quality is frequently reported.^175^ Previous studies have also documented the antimicrobial potential of silver nanoparticles against the seminal microflora.^176,177^ The antibacterial activity of LP@AgNPs may have contributed to reducing the microbial burden, resulting in the alleviation of microbial stress, protection of spermatozoa integrity, and consequently supporting improved semen quality.

The findings of this study are consistent with previous reports, which demonstrate that green-synthesized NPs can protect against reproductive damage, improve semen quality, and enhance overall reproductive health.^28,178–181^ While all these earlier studies primarily focused on testicular histopathology and selected biochemical markers. The present study adopts a more comprehensive approach by evaluating reproductive outcomes alongside their systemic safety and efficacy. Future studies should aim to elucidate the molecular mechanisms underlying each histopathological alteration, while establishing the translational safety and clinical potential of LP@AgNPs. A key priority will be given to long-term, chronic exposure and dose-dependent studies, coupled with comprehensive toxicokinetic, biodistribution, and multi-organ evaluations (including liver, kidney, reproductive organs, and blood) to establish their biosafety profile. The mechanistic investigations should further clarify the roles of oxidative stress, androgen-axis signaling, inflammation, and mitochondrial dysfunction. Reproductive functions must be evaluated through functional fertility trials (mating success and litter outcomes), advanced semen analysis (capacitation and acrosome reaction), and profiling of accessory gland secretory proteins. Additionally, studies on formulation behaviors in biological media, protein corona characterization, good laboratory practice (GLP)-compliant safety pharmacology, and allometric dose translation will be essential for advancing towards human applications.

## Conclusion

This study successfully demonstrated the phyto-mechanochemical synthesis of LP@AgNPs from LP aqueous extract and their therapeutic potential in alleviating male infertility. Comprehensive characterization of NPs confirmed their functional surface chemistry, biocompatibility, and structural integrity. The NPs exhibited an FCC crystalline structure, with an average crystallite size of 11.388 nm, were predominantly spherical, highly stable with a zeta potential of −41.18 mV, and had an average hydrodynamic diameter of 103.715 nm.

Given these structural properties, the study further evaluated the effects of LP and LP@AgNPs on reproductive parameters, vital organs, hormonal profiling, antioxidant enzyme activity, oxidative stress biomarkers, and semen quality in adult male Sprague-Dawley rats. The LP@AgNPs significantly improved the morphology of testicles and accessory glands, with notable increases in tubular dimensions, epithelial height, and spermatozoa accumulation, indicating enhanced spermatogenesis. Improved acinar organization in the prostate and epithelial architecture in seminal vesicles further supported enhanced functional activity. No signs of necrosis, bioaccumulation, or histopathological anomalies were observed in vital organs. Significant changes in plasma LH and T profiles were detected, and there was strict redox homeostasis, reflecting a balance between oxidant generation and antioxidant defense systems. Semen analysis revealed higher sperm counts, viability, and morphology in rats treated withLP@AgNPs. In contrast, the LP treatment alone produced relatively weaker effects, including prostatic acinar degeneration, epithelial thinning in seminal vesicles, and reduced sperm viability, underscoring its lower efficacy as a therapeutic agent relative to LP@AgNPs.

In summary, LP@AgNPs demonstrated promising potential in supporting male reproductive health by improving testicular function, enhancing the secretory epithelia of the accessory glands, preserving the structural integrity of vital organs, and promoting both hormonal and oxidative balance. While these findings highlight LP@AgNPs as a potential therapeutic candidate for male infertility, this study is preliminary and requires further validation. Future research should focus on elucidating the underlying molecular mechanisms, including the roles of kisspeptin and GnRH signaling, while establishing biosafety through dose-dependent toxicological profiling, long-term exposure studies, and functional fertility evaluations. Comprehensive pharmacokinetic, biodistribution, and translational investigations will be crucial before considering clinical applications.

## Ethical statement

This research study has been reviewed and is hereby approved (protocol #BEC-FBS-QAU2024-625) for implementation by the Bio-Ethical Committee (BEC) of Quaid-i-Azam University.

## Author contributions

Methodology, A. H., M. B. T., and S. J.; software, A. F., S. A.; formal analysis, M. M. A., A. M. A., S. J.; writing – original draft, A. H., and M. B. T.; writing – review and editing, M. B. T., S. J.; supervision, M. B. T., S. J.; project administration, M. B. T. All authors have read and agreed to the published version of the manuscript.

## Conflicts of interest

The authors declare that they have no conflicts of interest.

## Supplementary Material

RA-015-D5RA05660H-s001

## Data Availability

All data are present within the manuscript body. Supplementary information is available. See DOI: https://doi.org/10.1039/d5ra05660h.

## References

[cit1] Agarwal A., Saradha B., Neel P., Chak-Lam C., Ralf H., Sarah V., Mohamed A., Manesh Kumar P. S., Rupin S. (2021). Lancet.

[cit2] Agboola S., Ee K. Y., Huhn A. (2012). Food Chem..

[cit3] Pakpahan C., Agustinus A., Sa’adi A., Nguyen T. T. A., Liamputtong P., Effendy C., Hinting A. (2024). Heliyon.

[cit4] Lemma N., Sendo E. G., Abebe W. S. (2023). Inquiry.

[cit5] Leslie S. W. , Soon-Sutton T. L., Anu R. I., Hussain S. and Larry E. S., Prostate Cancer, StatPearls, Treasure Island, FL, USA, 2023

[cit6] Javaid S., Saima Walidad M., Erum J., Sarwat K., Asma J., Nidhi M. (2022). Prevalence of infertility and its causes in the population of Pakistan: A cross-sectional study. Ann. Rom. Soc. Cell Biol..

[cit7] Mustafa M. , Dar S. A., Azmi S. and Haque S., in. Oxidative Stress and Toxicity in Reproductive Biology and Medicine: A Comprehensive Update on Male Infertility Volume II, Springer, 2022, pp. 17–32

[cit8] Akang E. N., Opuwari C. S., Enyioma-Alozie S., Moungala L. W., Amatu T. E., Wada I., Ogbeche R. O., Ajayi O. O., Aderonmu M. M., Shote O. B. (2023). Sci. Rep..

[cit9] Okonofua F. E., Lorretta F. C. N., Akhere O., Oladiran A., Celestina O., Emmanuel U., Victor O. (2022). Int. J. Gen. Med..

[cit10] Dabaja A. A., Schlegel P. N. (2014). Transl. Androl. Urol..

[cit11] Iketubosin F. (2018). Gynaecology.

[cit12] Illingworth P. J., Venetis C., Gardner D. K., Nelson S. M., Berntsen J., Larman M. G., Agresta F., Ahitan S., Ahlström A., Cattrall F. (2024). Nat. Med..

[cit13] Zhang Y., Liu Y., Shen C., Guan Y. (2023). Sci. Rep..

[cit14] Ombelet W., Van Robays J. J. F. (2015). Views and V. I. Obgyn.

[cit15] Spanner E., de Graaf S., Rickard J. (2024). Anim. Reprod. Sci..

[cit16] Abdelkader A. M., Yeh J. J. O. (2009). G. International.

[cit17] Chronopoulou E., Gaetano-Gil A., Shaikh S., Raperport C., Al Wattar B. H., Ruiz-Calvo G., Zamora J., Bhide P. (2024). Acta Obstet. Gynecol. Scand..

[cit18] Chandy A. (2016). Current Medical Issues.

[cit19] Shiraishi E., Takae S., Faizal A. M., Sugimoto K., Okamoto A., Suzuki N. (2021). Int. J. Environ. Res. Public Health.

[cit20] Mertens J., Belva F., van Montfoort A. P., Regin M., Zambelli F., Seneca S., Couvreu de Deckersberg E., Bonduelle M., Tournaye H., Stouffs K. (2024). Nat. Commun..

[cit21] Fraser B., Peters A. E., Sutherland J. M., Liang M., Rebourcet D., Nixon B., Aitken R. J. (2021). Front. Physiol..

[cit22] Pritchard N., Kaitu’u-Lino T. U., Harris L., Tong S., Hannan N. (2021). Hum. Reprod. Update.

[cit23] Acharya B., Behera A., Behera S., Moharana S. (2024). ACS Appl. Bio Mater..

[cit24] Kumar V., Singh S., Srivastava B., Bhadouria R., Singh R. (2019). J. Environ. Chem. Eng..

[cit25] Nauroze T., Ali S., Kanwal L., Mughal T. A., Andleeb S., Ara C. (2023). Saudi J. Biol. Sci..

[cit26] Kiyimba K., Ahmed A., Choudhary M. I., Rehman K., Hasan S. M., Jabbar A., Obakiro S. B., Shah M. R., Munyendo W. L., Guantai E. M. (2025). PLoS One.

[cit27] Alwan S. H., Al-Saeed M. H. (2021). Biocatal. Agric. Biotechnol..

[cit28] Ali I. A. M., Ahmed A. B., Al-Ahmed H. I. (2023). Sci. Rep..

[cit29] Jebril S., Fdhila A., Dridi C. (2021). Sci. Rep..

[cit30] Barbir R., Capjak I., Crnković T., Debeljak Ž., Jurašin D. D., Ćurlin M., Šinko G., Weitner T., Vrček I. V. (2021). Chem. Biol. Interact..

[cit31] Irshad Z., Javaid S., Ali O. M., Khan M. S., AlSulami F. M. H., Almasoudi A., Al-Ghamdi A. A., Banbela H. M., Hajjar D., Makki A. A. (2025). ChemistrySelect.

[cit32] AlSulami F., Alsabban M. M., Banbela H. M., Zaidi N., Habib S., Hajjar D., Makki A. A., Bibi I., Javed T., Afzal A. (2025). Mater. Adv..

[cit33] Hamza A., Taj M. B., Jahan S., Ejaz S. A., Afzal A., Al Solami A. A., Zaidi N. (2025). New J. Chem..

[cit34] Mushtaq H., Batool I., Taj M. B., Ali O. M., Alnajeebi A. M., Yahya R., Alelwani W., Alsabban M. M., Alghamdi S. (2025). New J. Chem..

[cit35] Meissner H. O., Mscisz A., Kedzia B., Pisulewski P., Piatkowska E. (2015). Int. J. Biomed. Sci..

[cit36] Tarabasz D., Paweł S., Tomasz L., Wojciech P., Ewa B.-W., Dominik S., Wirginia K.-K., Henry O. M. (2020). Int. J. Mol. Sci..

[cit37] Fahoum M., Ross K. (2023). Curr. Res. Cmpl Alt. Med.

[cit38] Aroob S., Muhammad B. T., Saima S., Muhammad I., Raja H. A., Sadaf H., Ahmad R., Muhammad N. A., Muhammad A., Mika S. (2021). J. Environ. Chem. Eng..

[cit39] Santhi K., Sengottuvel R. (2016). Int. J. Curr. Microbiol. Appl. Sci..

[cit40] Garcia T., Lafuente D., Blanco J., Sánchez D. J., Sirvent J. J., Domingo J. L., Gómez M. (2016). Food Chem. Toxicol..

[cit41] Ul Haq M. N., Shah G. M., Gul A., Foudah A. I., Alqarni M. H., Yusufoglu H. S., Hussain M., Alkreathy H. M., Ullah I., Khan A. M. (2022). Nanomaterials.

[cit42] Hong M. Y., Seeram N. P., Zhang Y., Heber D. (2008). J. Med. Food.

[cit43] Vatanpour M., Ebrahimzadeh-Bideskan A., Rajabian A., Alipour F., Raoofi A., Ebrahimi V. (2024). Tissue Cell.

[cit44] Bouadi O. , Yao C., Zeng J., Beason D., Inda N., Malone Z., Yoshihara J., Manjally A. V., Johnson C., Cherry J., Chen C.-Y., Huang T.-C., Popovic B., Henley M., Liu G., Aichelman H., Davies S. W., Tian Y., Man H., Gilmore T., Ozsen E., Harder K., Walentek P., Kharitonova E. K., Zeldich E., Pitt D. and Tay T. L., bioRxiv, 2024, 10.1101/2024.07.26.605357

[cit45] Schindelin J., Arganda-Carreras I., Frise E., Kaynig V., Longair M., Pietzsch T., Preibisch S., Rueden C., Saalfeld S., Schmid B. (2012). Nat. Methods.

[cit46] Aebi H. , in Methods in Enzymology, Elsevier, 1984, vol. 105, pp. 121–126 10.1016/s0076-6879(84)05016-3 6727660

[cit47] Debnath D., Mandal T. K. (2000). J. Appl. Toxicol..

[cit48] Debnath D., Mandal T. K. (2000). J. Appl. Toxicol..

[cit49] Kumari R., Malaviya P. (2023). Environ. Sci. Pollut. Res..

[cit50] Ozcelebi H., Ari F., Dere E. (2021). Braz. Arch. Biol. Technol..

[cit51] Habig W. H., Pabst M. J., Jakoby W. B. (1974). J. Biol. Chem..

[cit52] Kaur R., Arora S. (2013). Free Radic. Biol. Med..

[cit53] Pütter J. , in Methods of Enzymatic Analysis, Elsevier, 1974, pp. 685–690

[cit54] Delom F., Mohtar M. A., Hupp T., Fessart D. (2020). Am. J. Physiol..

[cit55] Wright J., Colby H., Miles P. R. (1981). Biophysics.

[cit56] Iqbal M., Sharma S. D., Rezazadeh H., Hasan N., Abdulla M., Athar M. J. R. R. (1996). Redox Rep..

[cit57] Sivalingam A. M., Pandian A., Rengarajan S., Ramasubbu R., Parasuraman G., Sugumar V., Devaraj N. (2024). Biomass Convers. Biorefin..

[cit58] Hayashi I., Morishita Y., Imai K., Nakamura M., Nakachi K., Hayashi T. (2007). Mutat. Res., Genet. Toxicol. Environ. Mutagen..

[cit59] David M., Jahan S., Hussain J., Rehman H., Cloete K. J., Afsar T., Almajwal A., Alruwaili N. W., Razak S. (2022). Sci. Rep..

[cit60] Lorenzetti A., Marotta F., Yadav H., Celep G., Minelli E., Carrera Bastos P., Jain S., Polimeni A., Solimene U. (2012). Acta Bio-Med. Ateneo Parmense.

[cit61] Anous M., Abdalah E., Abdou A., El-Badawy A. (2017). Egypt. J. Anim. Prod..

[cit62] Mohamed M., Sulaiman S., Jaafar H., Sirajudeen K. (2012). Andrologia.

[cit63] Ngaha Njila M. I., Massoma Lembè D., Koloko B. L., Yong Meng G., Ebrahimi M., Awad E. A., Hasan Baiee F., Kenmogne H., Hambe M., Mandenguè S. H. (2019). Andrologia.

[cit64] Yelumalai S., Giribabu N., Karim K., Omar S. Z., Salleh N. B. (2019). Arch. Med. Sci..

[cit65] Kapourchali F. R., Louis X. L., Eskin M. N., Suh M. (2020). Birth Defects Res..

[cit66] Uzunhisarcikli M., Kalender Y., Dirican K., Kalender S., Ogutcu A., Buyukkomurcu F. (2007). Pestic. Biochem. Physiol..

[cit67] Zuhrotun A., Oktaviani D. J., Hasanah A. N. (2023). Molecules.

[cit68] Mikhailova E. O. (2024). Antibiotics.

[cit69] Chorosho S. H., Malik N., Panesar G., Kumari P., Jangra S., Kaur R., Al-Ghamdi M. S., Albishi T. S., Chopra H., Singh R. (2023). Oxid. Med. Cell. Longev..

[cit70] Ahmad T., Wani I. A., Manzoor N., Ahmed J., Asiri A. M. (2013). Colloids Surf., B.

[cit71] Malik M. A., Alshehri A. A., Patel R. (2021). J. Mater. Res. Technol..

[cit72] Wang L., Wu Y., Xie J., Wu S., Wu Z. (2018). Mater. Sci. Eng. C.

[cit73] Liaqat N., Jahan N., Anwar T., Qureshi H. (2022). Front. Chem..

[cit74] Shanker K., Mohan G. K., Hussain M. A., Jayarambabu N., Pravallika P. L. (2017). Pharmacogn. Mag..

[cit75] Gulzar N., Andleeb S., Raza A., Ali S., Liaqat I., Raja S. A., Ali N. M., Khan R., Awan U. A. (2024). Curr. Pharm. Biotechnol..

[cit76] Sziva R. E., Ács J., Tőkés A.-M., Korsós-Novák Á., Nádasy G. L., Ács N., Horváth P. G., Szabó A., Ke H., Horváth E. M. (2022). Life.

[cit77] Sengupta P., Nwagha U., Dutta S., Krajewska-Kulak E., Izuka E. (2017). Afr. Health Sci..

[cit78] Atli O., Baysal M., Aydogan-Kilic G., Kilic V., Ucarcan S., Karaduman B., Ilgin S. (2017). Asian J. Androl..

[cit79] Martin J. M., Bertram M. G., Saaristo M., Ecker T. E., Hannington S. L., Tanner J. L., Michelangeli M., O'Bryan M. K., Wong B. B. (2019). Sci. Total Environ..

[cit80] Santos R. A., Sousa A. P., Almeida-Santos T., Ramalho-Santos J., Tavares R. S. (2024). Asian J. Androl..

[cit81] Drobnis E. Z. , Nangia A. K., Drobnis E. Z. and Nangia A. K., in Impacts of Medications on Male Fertility, 2017, pp. 39–57

[cit82] Herzog B. J. , Nguyen H. M. T., Soubra A. and Hellstrom W. J., in Androgens: Clinical Research and Therapeutics, 2020, vol. 1, pp. 62–69

[cit83] Inhorn M. C., Patrizio P. (2015). Hum. Reprod. Update.

[cit84] Sharma S., Mittal S., Aggarwal P. (2009). BJOG Int. J. Obstet. Gynaecol..

[cit85] Ombelet W., Cooke I., Dyer S., Serour G., Devroey P. (2008). Hum. Reprod. Update.

[cit86] Barrera N. , Omolaoye T. S. and Du Plessis S. S., in. Fertility, Pregnancy, and Wellness, Elsevier, 2022, pp. 93–120

[cit87] Orihuela P. A. , in. Medical Plant Communications, 2022, vol. 5, pp. 11–15

[cit88] Gonzales G. F., Miranda S., Nieto J., Fernández G., Yucra S., Rubio J., Yi P., Gasco M. (2005). Reprod. Biol. Endocrinol..

[cit89] Ethiraj A. S., Jayanthi S., Ramalingam C., Banerjee C. (2016). Mater. Lett..

[cit90] Narayanan M., Divya S., Natarajan D., Senthil-Nathan S., Kandasamy S., Chinnathambi A., Alahmadi T. A., Pugazhendhi A. (2021). Process Biochem..

[cit91] Liem L. N., The N. P., Nguyen D. (2019). Technologies.

[cit92] Skow B. (2017). Philos. Sci..

[cit93] Wani I. A., Khatoon S., Ganguly A., Ahmed J., Ganguli A. K., Ahmad T. (2010). Mater. Res. Bull..

[cit94] Panda M. K., Dhal N. K., Kumar M., Mishra P. M., Behera R. K. (2021). Mater. Today: Proc..

[cit95] Gaddala B., Nataru S. (2015). Appl. Nanosci..

[cit96] Bharathi V., Shanthi S. (2017). World J. Pharm. Res..

[cit97] Rao Y. S., Kotakadi V. S., Prasad T., Reddy A. V., Gopal D. S. (2013). Spectrochim. Acta, Part A.

[cit98] Singh V., Shrivastava A., Wahi N. (2015). Afr. J. Biotechnol..

[cit99] Paosen S., Saising J., Septama A. W., Voravuthikunchai S. P. (2017). Mater. Lett..

[cit100] Sun Y., Dai C., Shi S., Zheng Y., Wei W., Cai D. (2018). J. Chromatogr. B.

[cit101] Korcan S. E., Kahraman T., Acikbas Y., Liman R., Ciğerci İ. H., Konuk M., Ocak İ. (2021). Microsc. Res. Tech..

[cit102] Supraja N., Avinash B., Prasad T. (2017). Int. J. Curr. Microbiol. Appl. Sci..

[cit103] Ahmad K., Noor R., Younus M., Chohan A., Asif H. M., Chishti A. W. (2020). RADS J. Pharm. Pharm. Sci..

[cit104] Shaban M., Mohamed F., Abdallah S. (2018). Sci. Rep..

[cit105] Kumar B., Smita K., Debut A., Cumbal L. (2020). Bioengineering.

[cit106] Juodenas M., Tamulevicius T., Henzie J., Erts D., Tamulevicius S. (2019). ACS Nano.

[cit107] Niwa E., Uematsu C., Hashimoto T. (2012). J. Am. Ceram. Soc..

[cit108] Zhang Q., Li N., Goebl J., Lu Z., Yin Y. (2011). J. Am. Chem. Soc..

[cit109] Meng F., Ding Y., Meng W., Mi G., Qiu J. (2020). ChemistrySelect.

[cit110] Smith M. A., Ilasi M. G., Zoelle A. (2013). J. Phys. Chem. C.

[cit111] Sklepova S. V., Ivanichok N., Kolkovskyi P., Kotsyubynsky V., Boychuk V., Rachiy B., Uhryński A., Bembenek M., Ropyak L. (2023). Materials.

[cit112] Luechinger N. A., Walt S. G., Stark W. J. (2010). Chem. Mater..

[cit113] Gonzales G. F., Vasquez V., Rodriguez D., Maldonado C., Mormontoy J., Portella J., Pajuelo M., Villegas L., Gasco M., Mathur P. (2007). Asian J. Androl..

[cit114] Gonzales G., Gasco M., Malheiros-Pereira A., Gonzales-Castañeda C. (2008). Andrologia.

[cit115] Tripathi U. K., Chhillar S., Kumaresan A., Aslam M. M., Rajak S., Nayak S., Manimaran A., Mohanty T., Yadav S. (2015). Vet. World.

[cit116] Kielbik P., Kaszewski J., Dabrowski S., Faundez R., Witkowski B. S., Wachnicki L., Zhydachevskyy Y., Sapierzynski R., Gajewski Z., Godlewski M. (2019). Nanotechnology.

[cit117] Ni D.-Q., Ma D.-D., Hao S.-L., Yang W.-X., Kovacs T., Tan F.-Q. (2021). Aging.

[cit118] Wang R., Song B., Wu J., Zhang Y., Chen A., Shao L. (2018). Int. J. Nanomed..

[cit119] Abu-Taweel G. M., Albetran H. M., Al-Mutary M. G., Ahmad M., Low I. M. (2021). Toxicol Rep.

[cit120] Omar C. A. (2018). Journal of Kerbala for Agricultural Sciences.

[cit121] Septiani R. A., Hamid I. S., Sabdoningrum E. K., Ma’ruf A., Hestianah E. P., Mafruchati M. (2022). Anim. Reprod. Sci..

[cit122] En Y. S., Primarizky H., Widjiati W., Luqman E. (2020). Iraqi J. Vet. Sci..

[cit123] Domesticada A. C. A. (2010). Int. J. Morphol..

[cit124] Hu X., Ouyang Q., Tang B., Zhang X., Hu J., Hu B., Hu S., Li L., He H., Liu H. (2022). Animals.

[cit125] Kováč J., Kročková J., Tvrdá E. (2019). J. Microbiol. Biotechnol. Food Sci..

[cit126] Hamood F. S. and Ahmad A. S., in. Journal of Pharmaceutical Negative Results, 2022, pp. 957–959

[cit127] de Oliveira G., Barcellos J., Lazzarini S., Rosas F. (2011). Anim. Biol..

[cit128] Kalynovskyi V., Pustovalov A., Grodzyuk G., Andriushyna N., Dzerzhynsky M. (2016). Regul. Mech. Biosyst..

[cit129] Nishino T., Wedel T., Schmitt O., Bühlmeyer K., Schönfelder M., Hirtreiter C., Schulz T., Kühnel W., Michna H. (2004). Ann. Anat..

[cit130] Mbyemeire H., Fasogbon I. V., Musyoka A. M., Oviosun A., Ojiakor V. O., Agunloye M. O., Wusa M., Okon M. B., Ikuomola E. O., Dangana R. S. (2025). F1000Research.

[cit131] Tarabasz D., Szczeblewski P., Laskowski T., Płaziński W., Baranowska-Wójcik E., Szwajgier D., Kukula-Koch W., Meissner H. O. (2022). Int. J. Mol. Sci..

[cit132] Zou Y., Aboshora W., Li J., Xiao T., Zhang L. (2017). Phytother Res..

[cit133] Shen Y., Fei X., Dai L., Zhu T., Liu J., Wu Z., Xu H., Li H., Wang Z. (2025). Gene Dis..

[cit134] Carrasco-Pozo C., Tan K. N., Rodriguez T., Avery V. M. (2019). Int. J. Mol. Sci..

[cit135] Hiipakka R. A., Zhang H.-Z., Dai W., Dai Q., Liao S. (2002). Biochem. Pharmacol..

[cit136] Paterni I., Granchi C., Katzenellenbogen J. A., Minutolo F. (2014). Steroids.

[cit137] Izumi K., Mizokami A., Lin W.-J., Lai K.-P., Chang C. (2013). Am. J. Pathol..

[cit138] Lund T. D., Munson D. J., Adlercreutz H., Handa R. J., Lephart E. D. (2004). Reprod. Biol. Endocrinol..

[cit139] Alex A. M., Subburaman S., Chauhan S., Ahuja V., Abdi G., Tarighat M. A. (2024). Sci. Rep..

[cit140] Selvam S., Wong Y. F., Tan J. S., Balakrishnan V., Thangeswaran D., Othman M. B. H., Ibrahim M. N. M., Jamil R., Hisham B. S. A., Raja P. B. (2025). Inorg. Chem. Commun..

[cit141] Tilbrook A., Clarke I. J. (2001). Biol. Reprod..

[cit142] Mbiydzenyuy N. E., Qulu L.-A. (2024). Metab. Brain Dis..

[cit143] El-Tantawy W. H., Temraz A., El-Gindi O. D. (2007). Int. Braz. J. Urol..

[cit144] JianFeng C., PengYing Z., ChengWei X., TaoTao H., YunGui B., KaoShan C. (2012). BMC Compl. Alternative Med..

[cit145] Oguebie R., Okeke C. (2023). GSJ.

[cit146] Sogorb M. A., Candela H., Estévez J., Vilanova E. (2023). Int. J. Mol. Sci..

[cit147] Fano D., Vásquez-Velásquez C., Gonzales-Castañeda C., Guajardo-Correa E., Orihuela P. A., Gonzales G. F. (2017). Evid. base Compl. Alternative Med..

[cit148] Aoki Y., Tsujimura A., Nagashima Y., Hiramatsu I., Uesaka Y., Nozaki T., Ogishima T., Shirai M., Shoyama Y., Tanaka H. (2019). Reprod. Med. Biol..

[cit149] Ohta Y., Yoshida K., Kamiya S., Kawate N., Takahashi M., Inaba T., Hatoya S., Morii H., Takahashi K., Ito M. (2016). Andrologia.

[cit150] Sultan A. R., Al-Kazazz F. F. M., Mohammed A. H. (2020). Nano Biomed. Eng..

[cit151] Mbiydzenyuy N. E., Qulu L.-A. (2024). Metab. Brain Dis..

[cit152] Yang Q., Jin W., Lv X., Dai P., Ao Y., Wu M., Deng W., Yu L. (2016). Pharm. Biol..

[cit153] Ibrahim R. M., Elmasry G. F., Refaey R. H., El-Shiekh R. A. (2022). ACS Omega.

[cit154] Wang S., Zhu F. (2019). Food Chem..

[cit155] Gan J., Feng Y., He Z., Li X., Zhang H. (2017). J. Food Qual..

[cit156] Ybañez-Julca R. O., Palacios J., Asunción-Alvarez D., Quispe-Díaz I., Nwokocha C. R., de Albuquerque R. D. D. G. (2022). Plant Foods Hum. Nutr..

[cit157] Derbak H., Imre K., Benabdelhak A. C., Moussaoui M., Kribeche A., Kebbi R., Ayad A. (2023). Vet. Sci..

[cit158] Inoue N., Farfan C., Gonzales G. (2016). Andrologia.

[cit159] Ye R.-J., Yang J.-M., Hai D.-M., Liu N., Ma L., Lan X.-B., Niu J.-G., Zheng P., Yu J.-Q. (2020). Fitoterapia.

[cit160] Tafuri S., Cocchia N., Vassetti A., Carotenuto D., Esposito L., Maruccio L., Avallone L., Ciani F. (2021). Nat. Prod. Res..

[cit161] Gasco M., Aguilar J., Gonzales G. (2007). Andrologia.

[cit162] Gonzales C., Rubio J., Gasco M., Nieto J., Yucra S., Gonzales G. F. (2006). J. Ethnopharmacol..

[cit163] Marcoccia D., Mollari M., Galli F. S., Cuva C., Tassinari V., Mantovani A. (2025). Reprod. Toxicol..

[cit164] Abou-Elghait A. T., Elgamal D. A., Abd el-Rady N. M., Hosny A., Abd El E. Z. A. A., Ali F. E. (2022). Tissue Cell.

[cit165] Hoxha M., Barbonetti A., Zappacosta B. (2023). Int. J. Mol. Sci..

[cit166] Verze P., Cai T., Lorenzetti S. (2016). Nat. Rev. Urol..

[cit167] Osadchuk L., Kleshchev M., Danilenko A., Osadchuk A. (2021). J. Trace Elem. Med. Biol..

[cit168] Chen Y., Wang X., Zhou J., Wang G., Gao T., Wei H., Che Y., Li T., Zhang Z., Wang S. (2024). Ecotoxicol. Environ. Saf..

[cit169] Marzec-Wróblewska U., Kamiński P., Łakota P., Szymański M., Wasilow K., Ludwikowski G., Kuligowska-Prusińska M., Odrowąż-Sypniewska G., Stuczyński T., Michałkiewicz J. (2011). Biol. Trace Elem. Res..

[cit170] Flores R., Angrimani D. d. S. R., Rui B. R., Brito M., Abreu R., Vannucchi C. I. (2017). Reprod. Domest. Anim..

[cit171] Plaszkó T., Szűcs Z., Vasas G., Gonda S. (2021). J. Fungi.

[cit172] Leiva-Revilla J., Cárdenas-Valencia I., Rubio J., Guerra-Castañón F., Olcese-Mori P., Gasco M., Gonzales G. (2012). Andrologia.

[cit173] Li G., Shen Q., Gao Y., Ma C., Song B., Wang C., Tang D., He X., Cao Y. (2024). Heliyon.

[cit174] Tvrdá E., Lovíšek D., Gálová E., Schwarzová M., Kováčiková E., Kunová S., Žiarovská J., Kačániová M. (2022). Int. J. Mol. Sci..

[cit175] Fijak M., Pilatz A., Hedger M. P., Nicolas N., Bhushan S., Michel V., Tung K. S., Schuppe H.-C., Meinhardt A. (2018). Hum. Reprod. Update.

[cit176] Pérez-Duran F., Acosta-Torres L. S., Serrano-Díaz P. N., Toscano-Torres I. A., Olivo-Zepeda I. B., García-Caxin E., Nuñez-Anita R. E. (2020). Syst. Biol. Reprod. Med..

[cit177] Tsakmakidis I. A., Samaras T., Anastasiadou S., Basioura A., Ntemka A., Michos I., Simeonidis K., Karagiannis I., Tsousis G., Angelakeris M. (2021). Animals.

[cit178] Mostafa-Hedeab G., Behairy A., Abd-Elhakim Y. M., Mohamed A. A.-R., Noreldin A. E., Dahran N., Gaber R. A., Alqahtani L. S., Essawi W. M., Eskandrani A. A. (2023). Antioxidants.

[cit179] Erfani Majd N., Hajirahimi A., Tabandeh M. R., Molaei R. (2021). Cell Tissue Res..

[cit180] Lyngdoh M. E., Chettri J., Kharchandy V. F., Sheel R., Choudhury A. R., Sarkar B., Pattanayak A., Deori S., Abedin S. N., Kadirvel G. (2024). Front. Bioeng. Biotechnol..

[cit181] Nauroze T., Ali S., Andleeb S., Ara C., Abbas A. S., Kanwal L., Mumtaz S., Hassan A., Ijaz F. (2025). Cell Biochem. Biophys..

