# Supplementary material for: Therapeutic effect of Lepidium peruvianum Chacon mediated silver nanoparticles on fertility in adult male Sprague-Dawley rats
Source: RSC Adv. 2025 Sep 17;15(41):34016–38. doi: 10.1039/d5ra05660h (PMC12442030; doi:10.1039/d5ra05660h)
Supplement: RA-015-D5RA05660H-s001 [file RA-015-D5RA05660H-s001.pdf]

Supplementary Information (SI) for RSC Advances.
This journal is © The Royal Society of Chemistry 2025

**Table S2** Effect of drug treatment on body weight gain and organ weights in experimental groups of adult Sprague Dawley rats				
	Treatment (µg/g per day)			
Parameters	Control	LP	LP@AgNPs	P Value
Initial body weight (g)	135.80 ± 3.48	143.60 ± 4.30	153.60 ± 2.32	
Final body weight (g)	197.20 ± 4.70	204.60 ± 3.49	210.40 ± 6.25	
Body weight gain (g)	61.40 ± 5.60	61.00 ± 5.54	56.80 ± 6.58	0.690
Testes weight (g)	1.07 ± 0.02	1.21 ± 0.08	1.14 ± 0.04	0.179
Epididymis weight (g)	0.53 ± 0.04	0.59 ± 0.04	0.51 ± 0.03	0.326
Seminal vesicles weight (g)	0.99 ± 0.09	1.19 ± 0.12	0.86 ± 0.07	0.079
Prostate weight (g)	0.62 ± 0.03	0.66 ± 0.01	0.58 ± 0.02	0.459
Liver weight (g)	4.37 ± 0.26	4.71 ± 0.27	4.86 ± 0.29	0.472
Kidney weight (g)	0.76 ± 0.02	0.73 ± 0.01	0.75 ± 0.04	0.743
Heart weight (g)	0.76 ± 0.01	0.79 ± 0.04	0.71 ± 0.02	0.246
Values are expressed as Mean ± SEM. Statistical Analysis: Analysis of variance (one way ANOVA followed by Tukey's multiple				

Supplementary Information

Therapeutic Potential of *Lepidium peruvianum Chacon* Mediated Silver Nanoparticles on Fertility in Adult Male Sprague-Dawley Rats

Tables

**Table S1** Elemental composition of LP@AgNPs based on EDX analysis		
Element	Weight %	Atomic %
CK	21.32	51.20
OK	5.62	17.99
NaK	2.14	3.57
KK	3.16	3.10
AgL	67.77	24.14
Total	100.00	

comparison test)

P value ≤ 0.05 is represented as "*"; P value ≤ 0.01 is represented as "**"; P value ≤ 0.001 is represented as "***"; P value ≤0.0001

is represented as "****".

"a" represents comparison of control with respective treatment groups while "b" represents comparison of the LP vs LP@AgNPs

Table S3 Effect sizes (Cohen's D and partial η²) for all the parameters across experimental groups

Parameter	Manuscript Reference	Group Comparison	Cohen's d	95 % Confidence Interval	Partial η²
Body weight gain	Table S2	Control vs. LP	0.051	–15.141to 15.943	0.059
Body weight gain	Table S2	Control vs. LP@AgNPs	0.420	–10.942 to 20.141	
Body weight gain	Table S2	LP vs. LP@AgNPs	0.490	–11.390 to 19.740	
Testes weight	Table S2	Control vs. LP	–1.131	–0.325 to 0.047	0.249
Testes weight	Table S2	Control vs. LP@AgNPs	–0.940	–0.247 to 0.125	
Testes weight	Table S2	LP vs.	–0.601	–0.108 to	

		LP@AgNPs		0.264	
Epididymis weight	Table S2	Control vs. LP	–0.554	–0.180 to 0.082	0.171
Epididymis weight	Table S2	Control vs. LP@AgNPs	0.370	–0.103 to 0.161	
Epididymis weight	Table S2	LP vs. LP@AgNPs	1.130	–0.055 to 0.209	
Seminal vesicles weight	Table S2	Control vs. LP	–0.840	–0.552 to 0.156	0.345
Seminal vesicles weight	Table S2	Control vs. LP@AgNPs	0.750	–0.220 to 0.488	
Seminal vesicles weight	Table S2	LP vs. LP@AgNPs	1.560	–0.022 to 0.686	
Prostate weight	Table S2	Control vs. LP	–0.800	–0.129 to 0.045	0.345
Prostate weight	Table S2	Control vs. LP@AgNPs	0.660	–0.047 to 0.127	
Prostate weight	Table S2	LP vs. LP@AgNPs	2.080	–0.005 to 0.169	
Liver weight	Table S2	Control vs. LP	–0.560	–1.385 to 0.7087	0.118
Liver weight	Table S2	Control vs. LP@AgNPs	–0.770	–1.531 to 0.563	
Liver weight	Table S2	LP vs. LP@AgNPs	–0.230	–1.193 to 0.900	
Kidney weight	Table S2	Control vs. LP	0.620	–0.077 to 0.133	0.048
Kidney weight	Table S2	Control vs.	0.040	–0.102 to	

		LP@AgNPs		0.108	
Kidney weight	Table S2	LP vs. LP@AgNPs	–0.400	–0.130 to 0.080	
Heart weight	Table S2	Control vs. LP	–0.380	–0.135 to 0.083	0.209
Heart weight	Table S2	Control vs. LP@AgNPs	1.011	–0.063 to 0.155	
Heart weight	Table S2	LP vs. LP@AgNPs	0.951	–0.037 to 0.180	
Seminiferous tubular EH	Fig. 4 (a)	Control vs. LP	–0.440	–2.275 to –0.380	0.090
Seminiferous tubular EH	Fig. 4 (a)	Control vs. LP@AgNPs	–0.750	–3.495 to –1.574	
Seminiferous tubular EH	Fig. 4 (a)	LP vs. LP@AgNPs	–0.340	–2.164 to –0.251	
Seminiferous tubular DD	Fig. 4 (b)	Control vs. LP	–0.280	–7.064 to –0.221	0.129
Seminiferous tubular DD	Fig. 4 (b)	Control vs. LP@AgNPs	–1.070	–14.48 to –7.543	
Seminiferous tubular DD	Fig. 4 (b)	LP vs. LP@AgNPs	–0.600	–10.81 to –3.925	
Seminiferous LD	Fig. 4 (c)	Control vs. LP	–0.040	–2.366 to 1.834	0.076
Seminiferous LD	Fig. 4 (c)	Control vs. LP@AgNPs	–0.650	–6.742 to –2.478	
Seminiferous LD	Fig. 4 (c)	LP vs. LP@AgNPs	–0.560	–6.464 to –2.223	
Seminiferous tubular area	Fig. 4 (d)	Control vs. LP	–0.350	–1172 to –129.4	0.144
Seminiferous tubular area	Fig. 4 (d)	Control vs. LP@AgNPs	–1.150	–2328 to –1271	
Seminiferous tubular area	Fig. 4 (d)	LP vs. LP@AgNPs	–0.580	–1673 to –624.900	
Caput EH	Fig. 6 (a)	Control vs. LP	–0.030	–0.634 to 0.560	0.370
Caput EH	Fig. 6 (a)	Control vs. LP@AgNPs	–1.390	–3.045 to –2.302	

Caput EH	Fig. 6 (a)	LP vs. LP@AgNPs	–1.650	–3.372 to –2.262	
Caput DD	Fig. 6 (b)	Control vs. LP	–0.530	–5.383 to –1.087	0.402
Caput DD	Fig. 6 (b)	Control vs. LP@AgNPs	–1.880	–14.840 to –10.530	
Caput DD	Fig. 6 (b)	LP vs. LP@AgNPs	–1.390	–11.600 to –7.301	
Caput LD	Fig. 6 (c)	Control vs. LP	–0.663	–5.447 to –1.402	0.133
Caput LD	Fig. 6 (c)	Control vs. LP@AgNPs	–0.930	–7.706 to –3.661	
Caput LD	Fig. 6 (c)	LP vs. LP@AgNPs	–0.338	–4.281 to –0.237	
Caput SMA	Fig. 6 (d)	Control vs. LP	–0.382	–428.50 to –48.20	0.167
Caput SMA	Fig. 6 (d)	Control vs. LP@AgNPs	–1.027	–955.40 to –571.40	
Caput SMA	Fig. 6 (d)	LP vs. LP@AgNPs	–0.696	–715.50 to –334.60	
Cauda EH	Fig. 6 (e)	Control vs. LP	–0.160	–0.758 to 0.264	0.300
Cauda EH	Fig. 6 (e)	Control vs. LP@AgNPs	–1.176	–2.537 to –1.513	
Cauda EH	Fig. 6 (e)	LP vs. LP@AgNPs	–1.047	–2.291 to –1.265	
Cauda DD	Fig. 6 (f)	Control vs. LP	–0.226	–7.756 to 1.163	0.067
Cauda DD	Fig. 6 (f)	Control vs. LP@AgNPs	–0.737	–13.15 to –4.275	
Cauda DD	Fig. 6 (f)	LP vs. LP@AgNPs	–0.383	–9.876 to –0.957	
Cauda LD	Fig. 6 (g)	Control vs. LP	–0.520	–11.550 to –2.626	0.083
Cauda LD	Fig. 6 (g)	Control vs. LP@AgNPs	–0.700	–14.140 to –5.219	
Cauda LD	Fig. 6 (g)	LP vs. LP@AgNPs	–0.190	–7.045 to 1.859	
Cauda SMA	Fig. 6 (h)	Control vs. LP	–0.113	–854 to 357.6	0.054
Cauda SMA	Fig. 6 (h)	Control vs. LP@AgNPs	–0.518	–1832 to –624.1	

Cauda SMA	Fig. 6 (h)	LP vs. LP@AgNPs	–0.448	–1585 to –375	
Prostate EH	Fig. 8 (a)	Control vs. LP	1.080	1.570 to 2.854	0.530
Prostate EH	Fig. 8 (a)	Control vs. LP@AgNPs	–1.515	–5.394 to –4.110	
Prostate EH	Fig. 8 (a)	LP vs. LP@AgNPs	–2.400	–7.606 to –6.322	
Prostate DD	Fig. 8 (b)	Control vs. LP	0.02	–14.100 to 14.870	0.05
Prostate DD	Fig. 8 (b)	Control vs. LP@AgNPs	–0.52	–23.030 to 5.939	
Prostate DD	Fig. 8 (b)	LP vs. LP@AgNPs	–0.440	–23.420 to 5.553	
Prostate LD	Fig. 8 (c)	Control vs. LP	–0.053	–12.800 to 11.060	0.042
Prostate LD	Fig. 8 (c)	Control vs. LP@AgNPs	–0.49	–19.070 to 4.796	
Prostate LD	Fig. 8 (c)	LP vs. LP@AgNPs	–0.36	–18.200 to 5.665	
FSH	Fig. 11 (a)	Control vs. LP	–1.17	–1.371 to 0.335	0.239
FSH	Fig. 11 (a)	Control vs. LP@AgNPs	0.069	–0.817 to 0.889	
FSH	Fig. 11 (a)	LP vs. LP@AgNPs	1.010	–0.299 to 1.407	
LH	Fig. 11 (b)	Control vs. LP	1.650	–0.084 to 1.492	0.668
LH	Fig. 11 (b)	Control vs. LP@AgNPs	2.840	0.662 to 2.238	
LH	Fig. 11 (b)	LP vs. LP@AgNPs	1.620	–0.042 to 1.534	
T	Fig. 11 (c)	Control vs. LP	–3.950	–7.972 to –0.578	0.790
T	Fig. 11 (c)	Control vs. LP@AgNPs	–3.780	–13.001 to –5.611	
T	Fig. 11 (c)	LP vs. LP@AgNPs	–1.880	–8.730 to –1.336	
Catalase	Fig. 11 (d)	Control vs. LP	0.600	–18.690 to 40.280	0.111
Catalase	Fig. 11 (d)	Control vs. LP@AgNPs	–0.077	–31.100 to 27.860	
Catalase	Fig. 11 (d)	LP vs.	–0.990	–41.890 to	

		LP@AgNPs		17.070	
POD	Fig. 11 (e)	Control vs. LP	0.350	–0.248 to 0.351	0.249
POD	Fig. 11 (e)	Control vs. LP@AgNPs	–0.980	–0.462 to 0.1365	
POD	Fig. 11 (e)	LP vs. LP@AgNPs	–1.010	–0.513 to 0.085	
GST	Fig. 11 (f)	Control vs. LP	0.190	–0.668 to 0.851	0.059
GST	Fig. 11 (f)	Control vs. LP@AgNPs	–0.420	–0.911 to 0.607	
GST	Fig. 11 (f)	LP vs. LP@AgNPs	–0.500	–1.003 to 0.516	
GPx	Fig. 11 (g)	Control vs. LP	–0.300	–1.059 to 0.6920	0.112
GPx	Fig. 11 (g)	Control vs. LP@AgNPs	0.380	–0.656 to 1.095	
GPx	Fig. 11 (g)	LP vs. LP@AgNPs	1.230	–0.472 to 1.279	
GSR	Fig. 11 (h)	Control vs. LP	0.400	–1.277 to 1.925	0.027
GSR	Fig. 11 (h)	Control vs. LP@AgNPs	0.261	–1.335 to 1.867	
GSR	Fig. 11 (h)	LP vs. LP@AgNPs	–0.057	–1.659 to1.543	
MDA	Fig. 11 (i)	Control vs. LP	–0.751	–1.185 to 0.752	0.207
MDA	Fig. 11 (i)	Control vs. LP@AgNPs	–0.970	–1.602 to 0.336	
MDA	Fig. 11 (i)	LP vs. LP@AgNPs	–0.602	–1.386 to 0.552	
ROS	Fig. 11 (j)	Control vs. LP	–0.420	–0.285 to 0.157	0.067
ROS	Fig. 11 (j)	Control vs. LP@AgNPs	–0.560	–0.289 to 0.152	
ROS	Fig. 11 (j)	LP vs. LP@AgNPs	–0.043	–0.226 to 0.216	
Spermatozoa Count	Fig. 12 (a)	Control vs. LP	–3.040	–70.180 to –16.220	0.970
Spermatozoa Count	Fig. 12 (a)	Control vs. LP@AgNPs	–10.130	–217.800 to –163.800	
Spermatozoa	Fig. 12 (a)	LP vs.	–10.210	–174.600 to	

Count		LP@AgNPs		–120.600	
Spermatozoa viability	Fig. 12 (b)	Control vs. LP	2.702	5.879 to 20.920	0.853
Spermatozoa viability	Fig. 12 (b)	Control vs. LP@AgNPs	–2.390	–17.520 to –2.479	
Spermatozoa viability	Fig. 12 (b)	LP vs. LP@AgNPs	–5.611	–30.920 to –15.88	
Spermatozoa morphology	Fig. 12 (c)	Control vs. LP	2.654	3.206 to 16.531	0.876
Spermatozoa morphology	Fig. 12 (c)	Control vs. LP@AgNPs	–3.031	–19.662 to –6.399	
Spermatozoa morphology	Fig. 12 (c)	LP vs. LP@AgNPs	–6.020	–29.531 to –16.212	
Spermatozoa motility	Fig. 12 (d)	Control vs. LP	2.563	8.185 to 28.380	0.803
Spermatozoa motility	Fig. 12 (d)	Control vs. LP@AgNPs	–4.080	–16.150 to 3.046	
Spermatozoa motility	Fig. 12 (d)	LP vs. LP@AgNPs	–3.582	–33.940 to –14.741	
Note: A negative sign in Cohen's d indicates that the mean of the second group is greater than that of the first group in the calculation.					

Table S4. Comparative evaluation of AgNP synthesis methods: yield, average particle size, reproducibility, and scalability of the phyto-mechanochemical approach (this work) versus conventional phytosynthesis, chemical reduction, and sol–gel methods.

Method	Percentage yield of $Ag^+ \rightarrow$ AgNPs)	Avg. Particle Size (nm)	Std. Dev. (Reproducibility, n=3)	Scalability Potential
Phyto-mechanochemical (this work)	89 % ± 2.1	12 ± 3	±1.8 nm	High (tested up to 10×)
Conventional phytosynthesis	72 % ± 3.5	25 ± 5	±4.2 nm	Moderate
Chemical reduction ($NaBH_4$)	95 % ± 1.2	12 ± 2	±1.0 nm	High
Thermal/sol-gel method	80 % ± 2.8	20 ± 4	±3.6 nm	Low (energy intensive)

Supplementary Figures

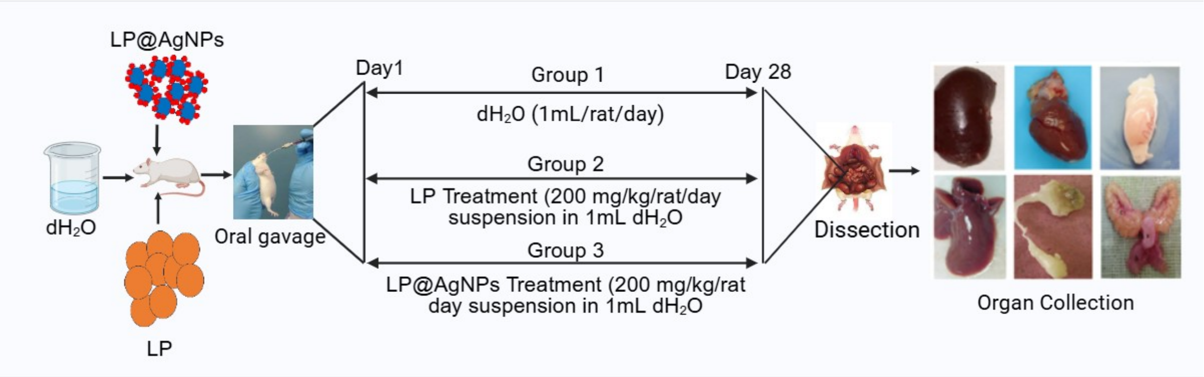

Fig. S1 Schematic diagram of experimental design for evaluating the effects of LP and LP@AgNPs on reproductive health in adult Sprague Dawley rats after 28 days of treatment exposure**.**

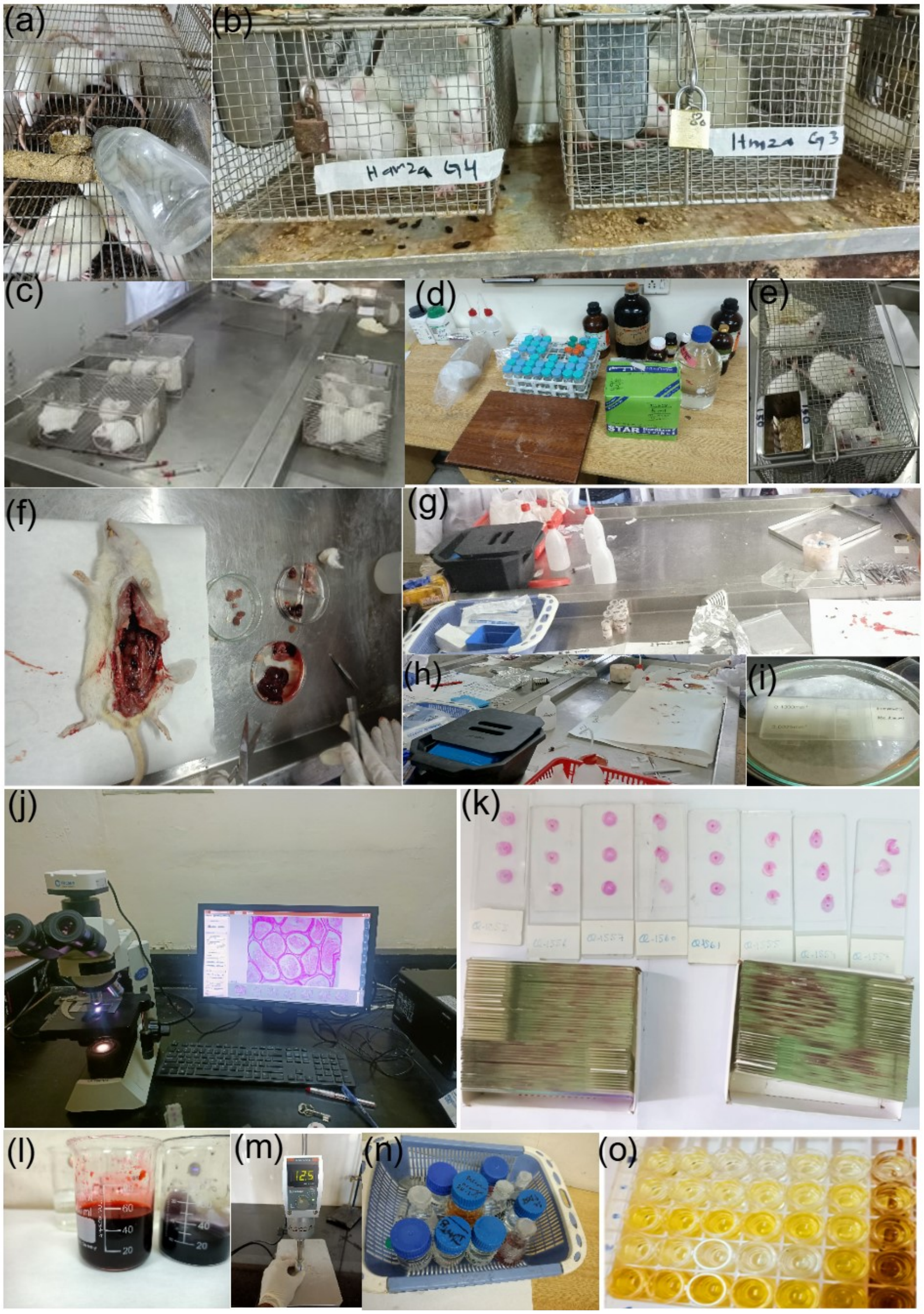

Fig. S2: Detailed morphological alterations in experimental animals and representative macroscopic images; (a) animal acclimatization, (b) Rats on the first day of dosage exposure (c) Dose administration or dissection bench,(d) Animal dissection preparations, (e) LM@AgNPs few minutes before dissections, (f) Dissection and organ collection, (g,h) after dissection, (i) Improved

Neubauer chamber, (j) microscopy, (k) Histopathological slides analyzed, (l) H & E stain, (m) Homogenizer, (n) Antioxidant solutions, (o) Enzyme linked immunosorbent assay kit

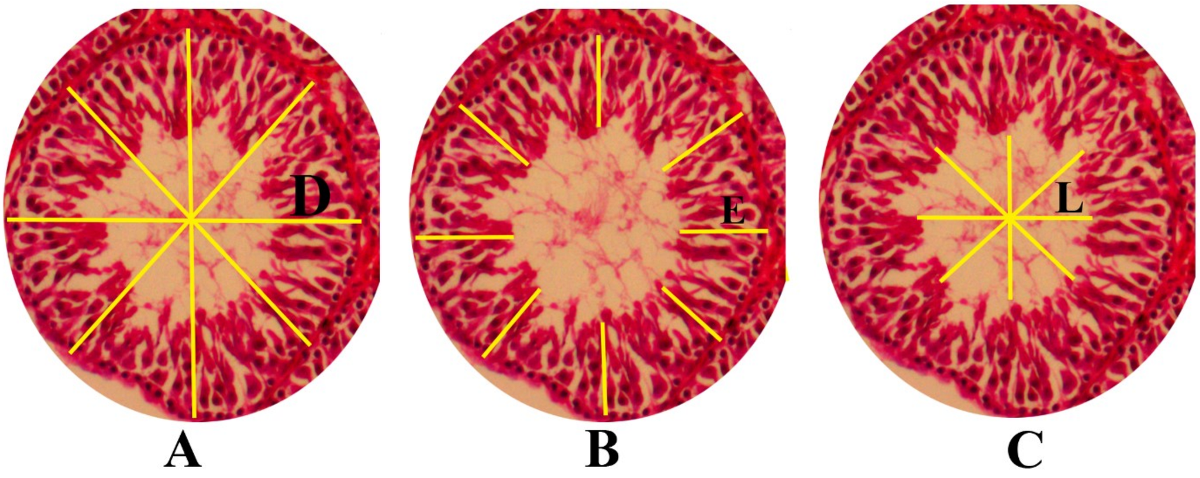

Fig. S3 Histomorphometric analysis of seminiferous tubules using Figi ImageJ software. Representative cross sections of seminiferous tubules (A-C) highlights morphometric measurements. Yellow lines indicate parameters: seminiferous tubules diameters (D), Lumen diameters (L), and epithelial thickness (E). In panel A, the average of all measurements for each parameter was calculated and treated as a single value; a procedure similarly applied to the panels B and C. Measurements were recorded using straight/segmented and free hand lines tool of Fiji Image J software for accurate quantitative analysis. All the measurements were taken at X100 magnification and the scale is mentioned in supplementary figure 4 for reproducibility.

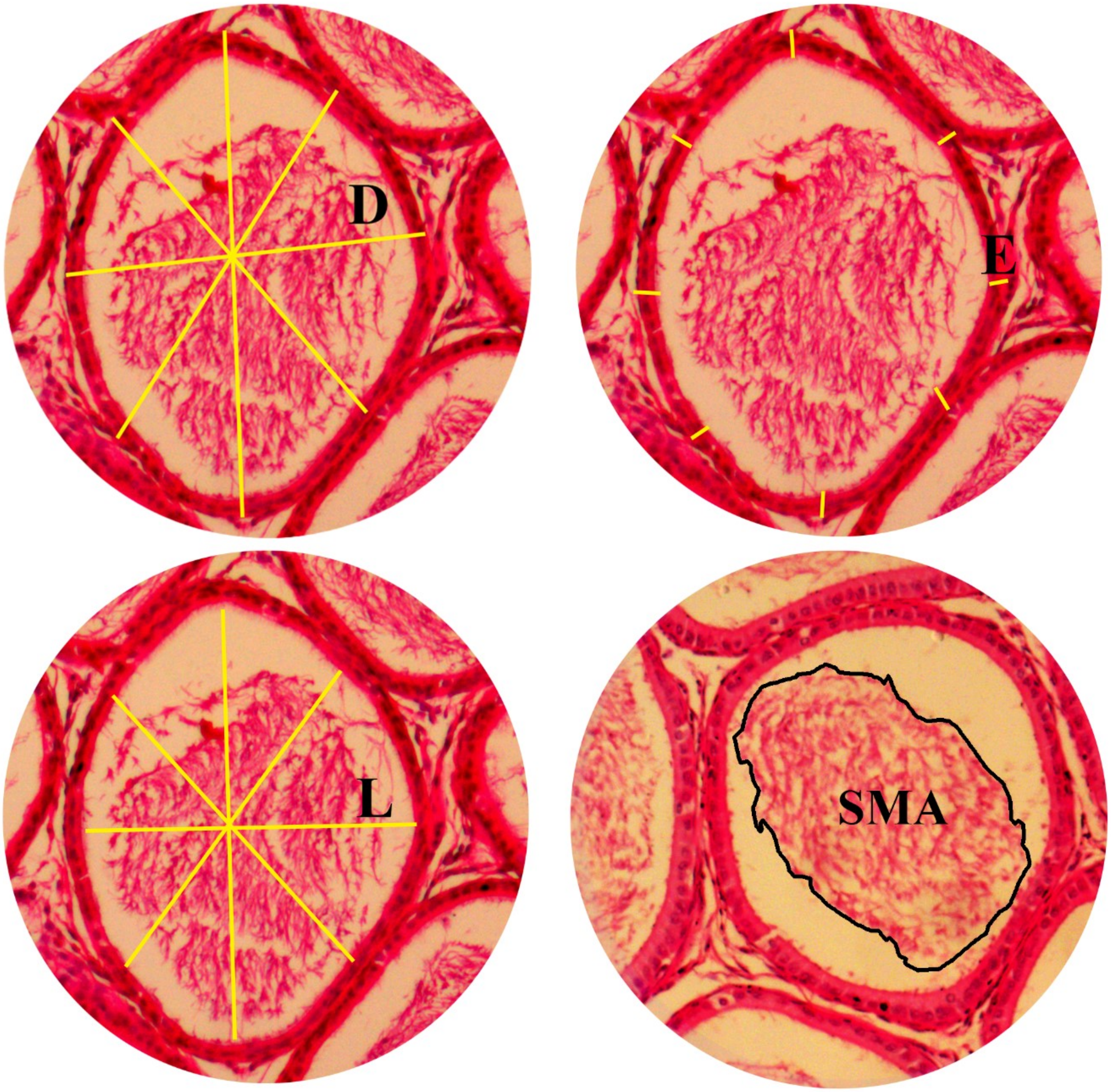

Fig. S4 Histomorphometric analysis of epididymis using Figi ImageJ. Representative cross sections of epididymal tubules are demonstrating morphometric measurements. Yellow lines indicate the parameters analyzed: tubular diameter (D), epithelial thickness (E) and lumen diameter (L). The sperm mass area (SMA) is outlined in black. Measurements in all panels were averaged and recorded as a single value for each parameter. Measurements were recorded using straight/segmented/ free hand lines and free hand selection tools of Fiji Image J software for accurate quantitative analysis. All the measurements were taken at X100 magnification and the scale is mentioned in supplementary figure 5-6 for reproducibility.

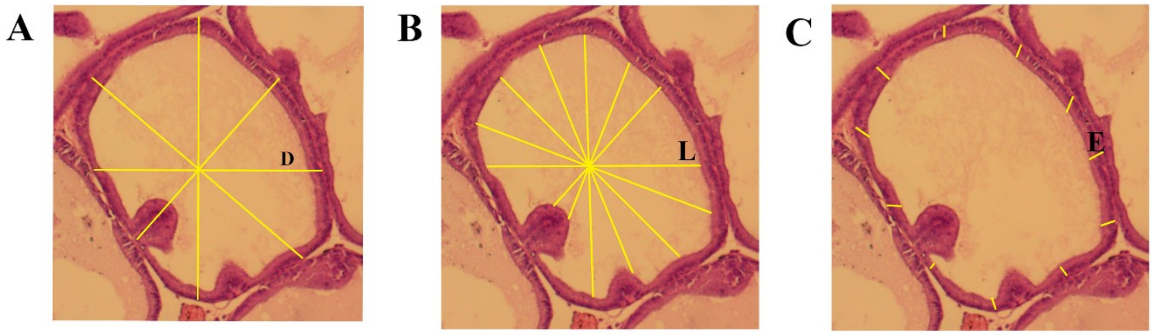

Fig. S5 Histomorphometric analysis of ventral prostate using Figi ImageJ. Representative cross sections (A-C) of prostate tubules are demonstrating morphometric measurements. The yellow lines indicate the parameters analyzed: tubular diameter (D), lumen diameter (L) and epithelial thickness (E). Measurements in all panels were averaged and recorded as a single value for each parameter. Measurements were recorded using straight/segmented/ free hand lines tool of Fiji Image J software for accurate quantitative analysis. All the measurements were taken at X100 magnification and the scale is mentioned in supplementary figure 7 for reproducibility.

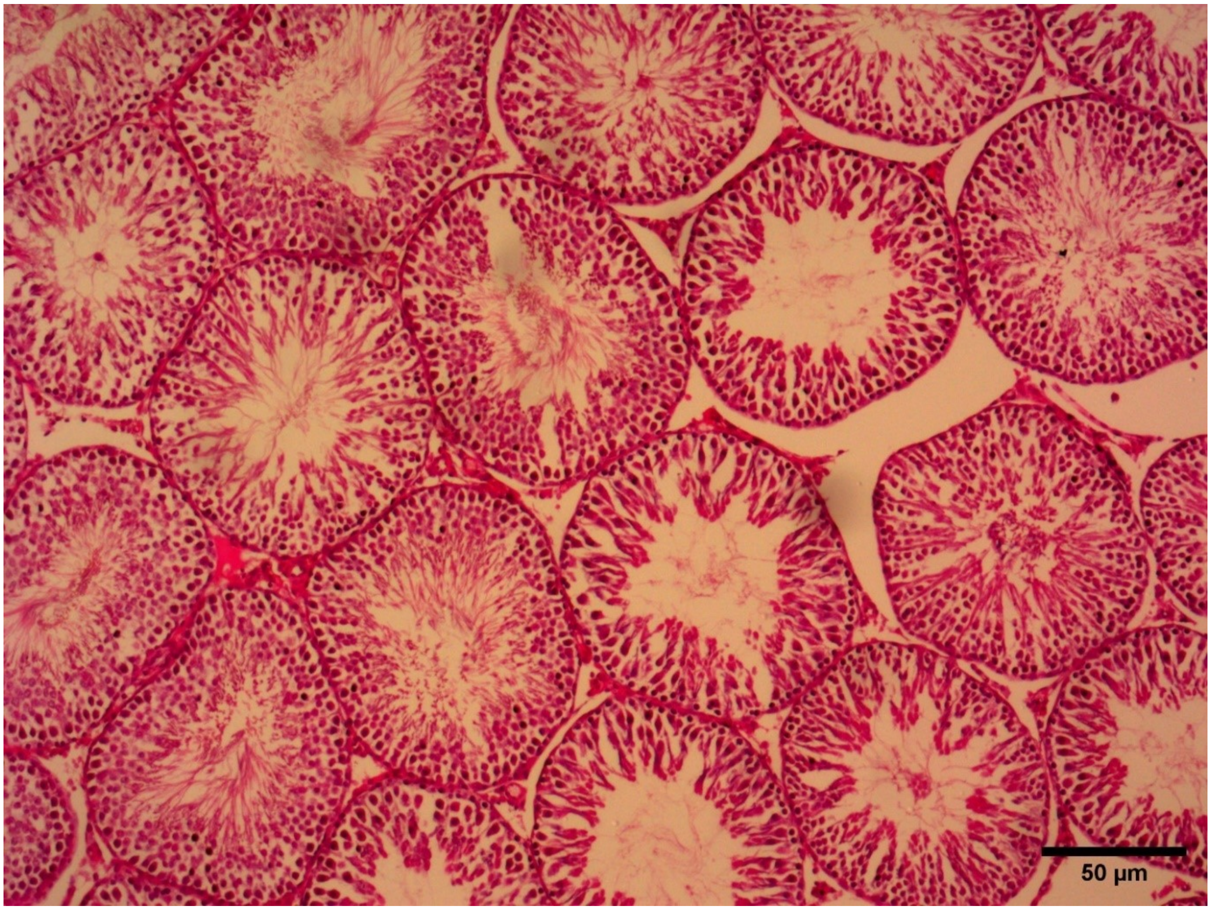

Fig. S6 Scaled histological image of testes tissues at X100 magnification**.** The image shows seminiferous tubule stained with H & E, highlighting structural organization and cellular detail. The scale bar represents 50 µm calibrated for morphometric analyses across histological evaluations.

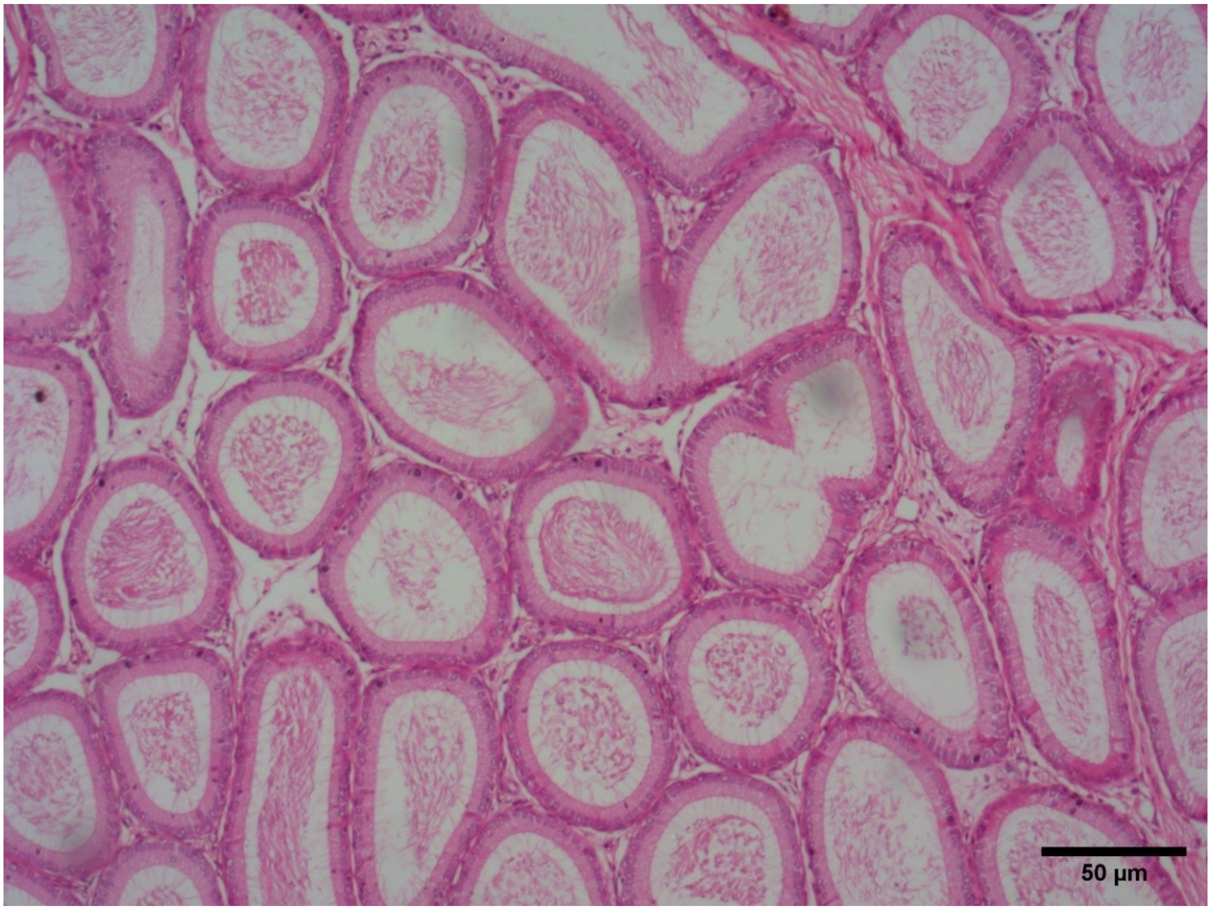

Fig. S7 Scaled histological image of caput epididymis tissues at 100X magnification. The image shows caput tubule stained with H & E, highlighting structural organization and cellular detail. The scale bar represents 50 μm calibrated for morphometric analyses across histological evaluations.

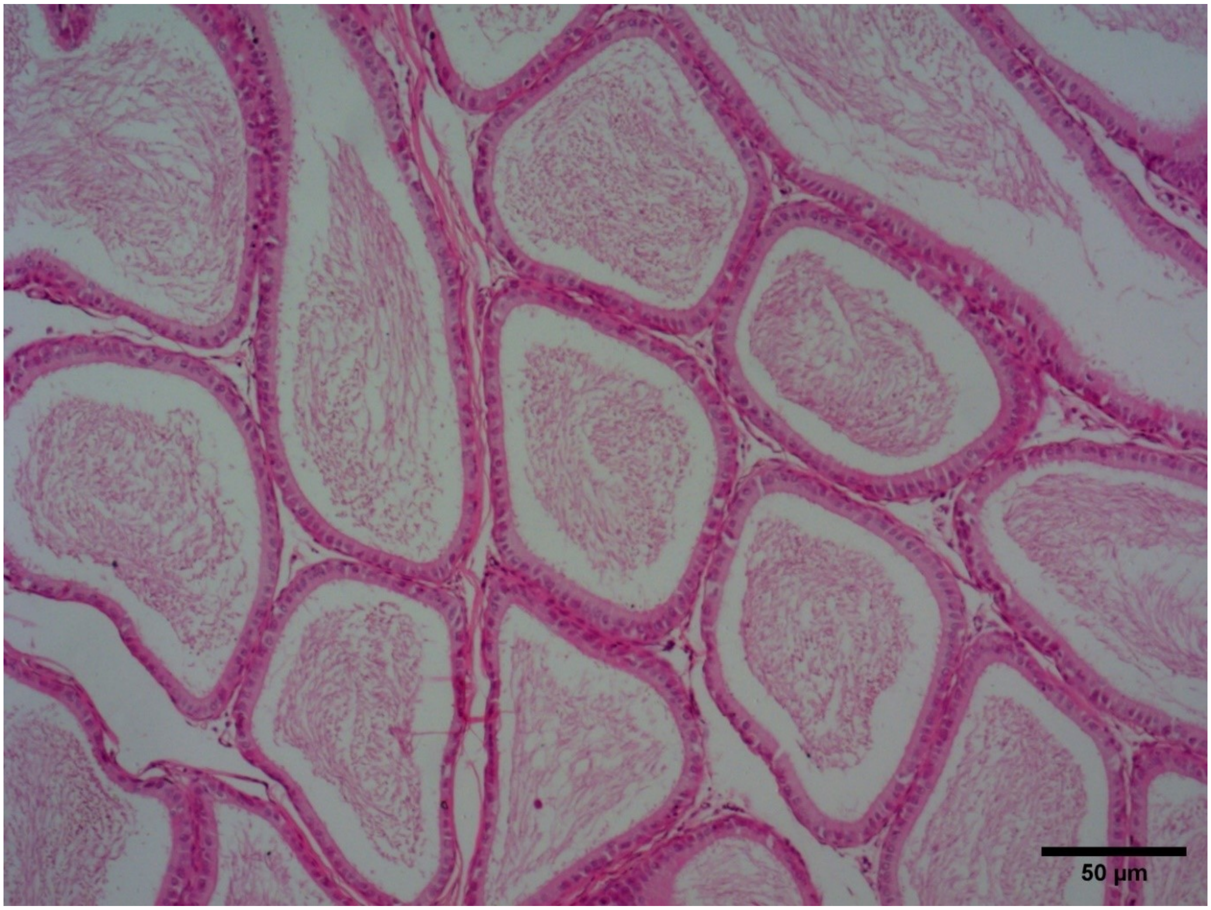

Fig. S8 Scaled histological image of cauda epididymis tissues at X100 magnification**.** The image shows cauda tubules stained with H & E, highlighting structural organization and cellular detail. The scale bar represents 50 µm calibrated for morphometric analyses across histological evaluations.

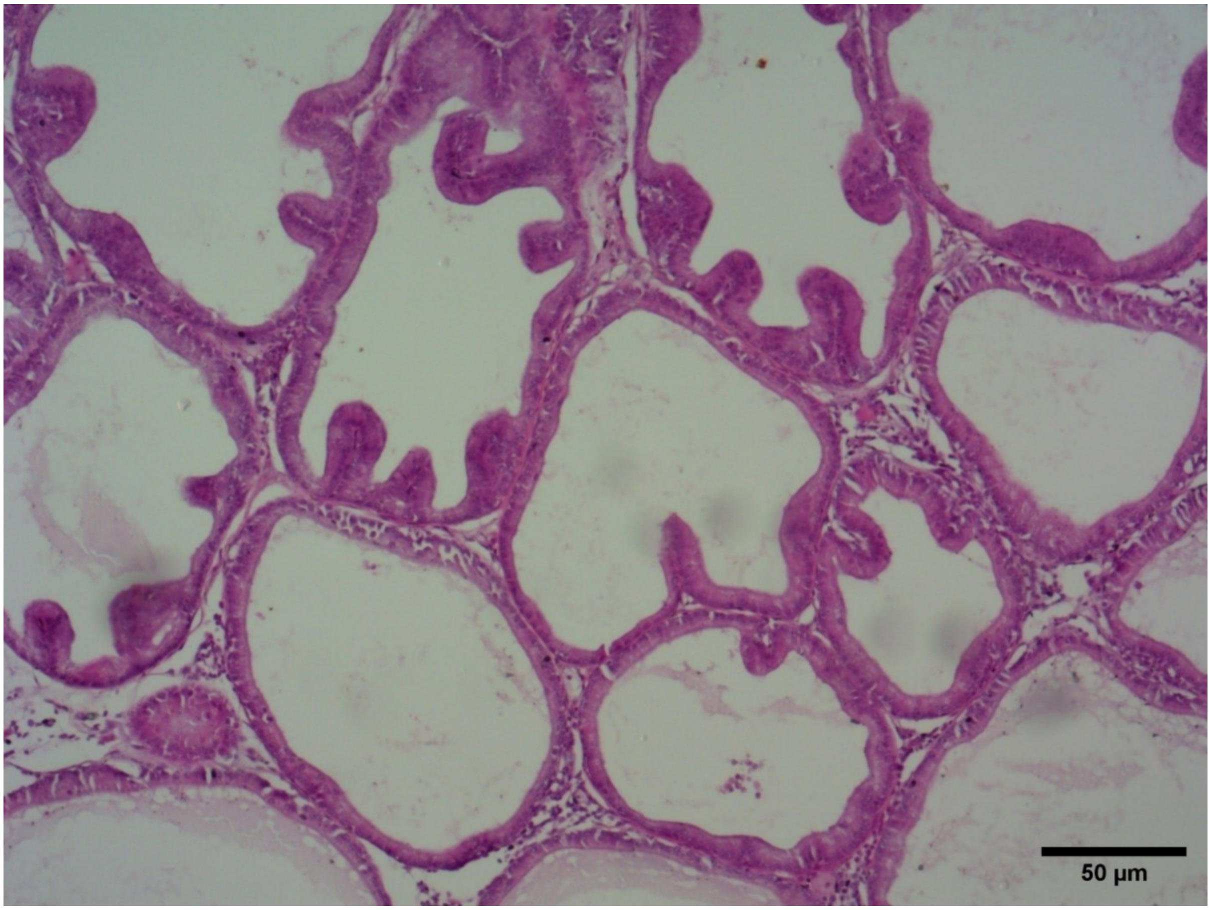

Fig. S9 Scaled histological image of ventral prostate tissues at X100 magnification. The image shows prostate tubules stained with H & E, highlighting structural organization and cellular detail. The scale bar represents 50 µm calibrated for morphometric analyses across histological evaluations

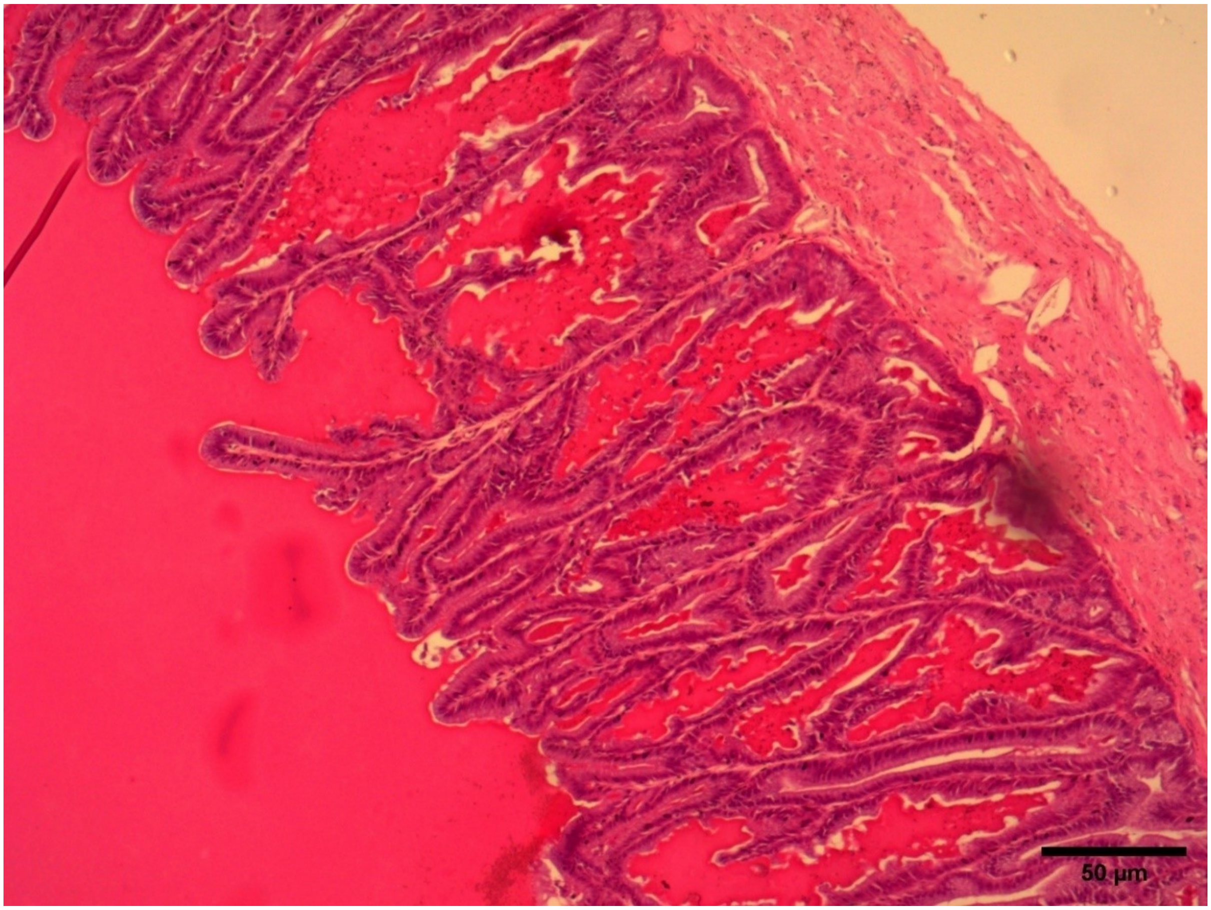

Fig. S10 Scaled histological image of seminal vesicles tissues at X100 magnification. The image shows seminal vesicles stained with H & E, highlighting structural organization and cellular detail. The scale bar represents 50 µm calibrated for morphometric analyses across histological evaluations.

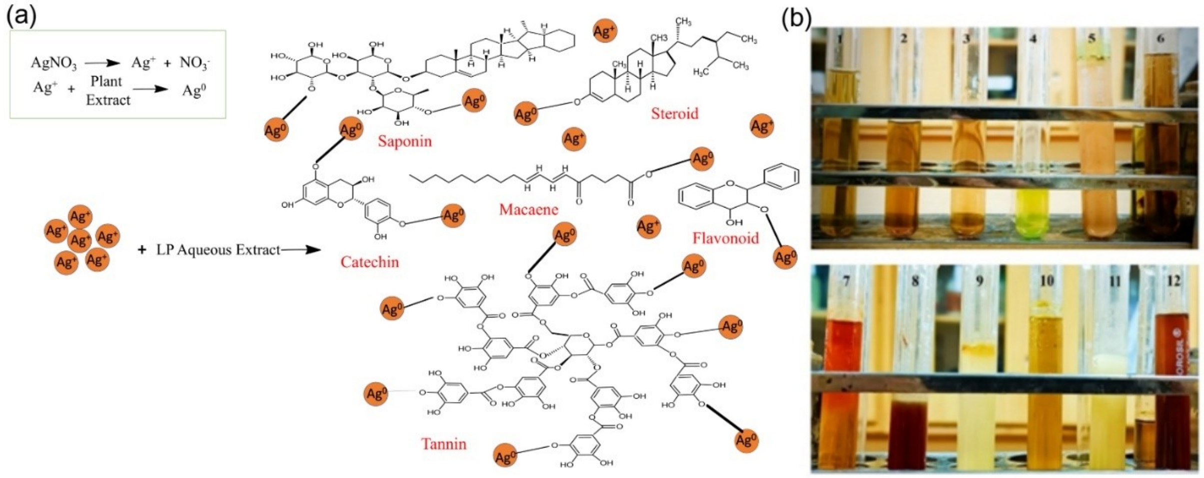

Fig. S11 The proposed reduction scheme for LP@AgNPs synthesis, (b) Representative picture for the phytochemical screening of LP extracts; 1: tannins 2: flavonoids 4: polyphenols 5: anthraquinone 7: steroids 9: saponin 10: coumarin 11: alkaloids 12: glycosides

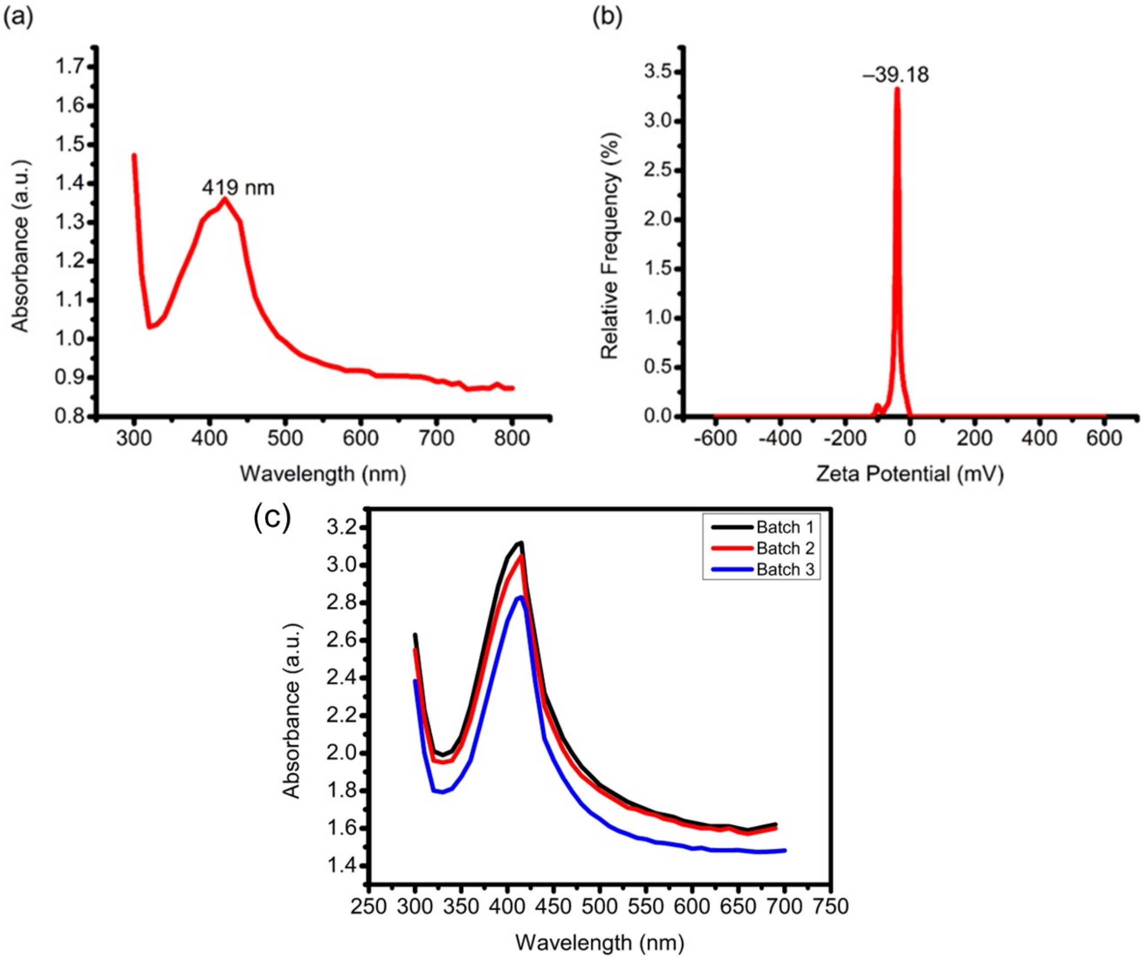

Fig. S12 (a) UV-Vis absorption spectrum of LP@AgNPs recorded one year post-synthesis confirmed their long term storage stability. (b) Zeta potential analysis confirmed a surface charge of –39.18, indicated high colloidal stability that pevented agglomeration over time. (c) Reproducibility of LP@AgNPs via phytomechanochemical route

Workbook name	Untitled1.apkw		
Measurement name	N4-Ameer - NPs-Size 3		
Measurement mode	Particle size		
**Comment**			
**Expert advices**			
**Results**	Hydrodynamic diameter	103.7153021	nm
	Polydispersity index	0.279387132	
	Intercept $g1^2$	0.603330337	
	Baseline	1.000551445	
	Mean intensity	288.2464646	kcps
	Absolute intensity	191597.0911	kcps
	Fit error	0.000227135	
	Diffusion coefficient	4.730027308	$\mu m^2/s$
Peak analysis volume	Peak volume 1	5.475849156	nm
	Area volume 1	100.00	%
	Standard deviation volume 1	1.60996	nm
	Peak volume 2		
	Area volume 2		
	Standard deviation volume 2		
	Peak volume 3		
	Undersize span intensity (D90-D10)/D50	1.991494523	
	Undersize span number (D90-D10)/D50	0.947507351	
**Automatic values**	Filter optical density	2.822624922	
	Focus position	-3.702947855	mm
	Auto run criteria	173.1509	%
	Transmittance	44.59021688	%
	Angle used	BackScatter	
	Processed runs	6	
**Input parameters**	**General**		
	Sample id		
	Batch number		
	Measurement cell	Disposable	
	Measurement angle	BackScatter	
	Target temperature	25	°C
	Equilibration time	0h 00m 01s	
	Analysis model	General	
	Cumulant model	Advanced	

Additional information	Measurement	
	User	Hp
	Start time	10/18/2024 16:56
	Software version	3.2.5
	Computer name	DESKTOP-0179VA6
	**Instrument**	
	Type	Litesizer DLS 700
	Serial number	84665652
	**Module**	
	Type	FM10
	Serial number	84571090

Fig. S13 Original machine generated values of DLS chracterization

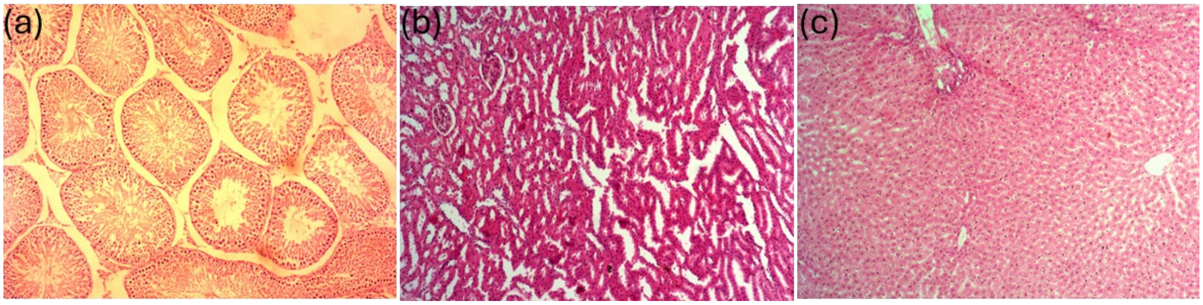

Fig. S14 Photomicrographs of testes, kidney and liver following acute exposure to 1000 mg/kg rat body weight of LM@AgNPs. No histopathological abnormalities were observed on day 14
